# Adverse effects of **Δ**^9^-tetrahydrocannabinol on neuronal bioenergetics during postnatal development

**DOI:** 10.1172/jci.insight.135418

**Published:** 2020-12-03

**Authors:** Johannes Beiersdorf, Zsofia Hevesi, Daniela Calvigioni, Jakob Pyszkowski, Roman Romanov, Edit Szodorai, Gert Lubec, Sally Shirran, Catherine H. Botting, Siegfried Kasper, Geoffrey W. Guy, Roy Gray, Vincenzo Di Marzo, Tibor Harkany, Erik Keimpema

**Affiliations:** 1 Department of Molecular Neurosciences, Center for Brain Research, Medical University of Vienna, Vienna, Austria.; 2 Optics11, VU University Campus, Amsterdam, Netherlands.; 3 Paracelsus Private Medical University, Salzburg, Austria.; 4 School of Chemistry, University of St. Andrews, St. Andrews, United Kingdom.; 5 Department of Psychiatry and Psychotherapy, Medical University of Vienna, Vienna, Austria.; 6 GW Phamaceuticals, Salisbury, Wiltshire, United Kingdom.; 7 Endocannabinoid Research Group, Institute of Biomolecular Chemistry, Consiglio Nazionale delle Ricerche, Pozzuoli, Italy.; 8 Canada Excellence Research Chair, Institut Universitaire de Cardiologie et de Pneumologie de Québec and Institut sur la Nutrition et les Aliments Fonctionnels, Université Laval, Québec, Québec, Canada.; 9 Department of Neuroscience, Biomedikum D7, Karolinska Institutet, Solna, Sweden.

**Keywords:** Cell Biology, Development, Neurodevelopment

## Abstract

Ongoing societal changes in views on the medical and recreational roles of cannabis increased the use of concentrated plant extracts with a Δ^9^-tetrahydrocannabinol (THC) content of more than 90%. Even though prenatal THC exposure is widely considered adverse for neuronal development, equivalent experimental data for young age cohorts are largely lacking. Here, we administered plant-derived THC (1 or 5 mg/kg) to mice daily during P5–P16 and P5–P35 and monitored its effects on hippocampal neuronal survival and specification by high-resolution imaging and iTRAQ proteomics, respectively. We found that THC indiscriminately affects pyramidal cells and both cannabinoid receptor 1^+^ (CB_1_R)^+^ and CB_1_R^–^ interneurons by P16. THC particularly disrupted the expression of mitochondrial proteins (complexes I–IV), a change that had persisted even 4 months after the end of drug exposure. This was reflected by a THC-induced loss of membrane integrity occluding mitochondrial respiration and could be partially or completely rescued by pH stabilization, antioxidants, bypassed glycolysis, and targeting either mitochondrial soluble adenylyl cyclase or the mitochondrial voltage-dependent anion channel. Overall, THC exposure during infancy induces significant and long-lasting reorganization of neuronal circuits through mechanisms that, in large part, render cellular bioenergetics insufficient to sustain key developmental processes in otherwise healthy neurons.

## Abstract 2

Repeated THC exposure in juvenile mice compromises the limbic circuitry, with life-long impairment to the respiration of neurons.

## Introduction

The ongoing legalization of cannabis products triggered a sea change in preference to preparations with high psychoactive potency, with some oils and waxes by now reaching more than 90% of Δ^9^-tetrahydrocannabinol (THC) content (1, 2). These trends, together with the increased accessibility of THC-containing products to even young age groups, suggest that, besides adverse metabolic symptoms in adults (hypertension, hyperthermia, tachycardia, cardiotoxicity; ref. 3), THC could also be neurotoxic (or neuromodulatory) if taken during prepuberty (4). This consideration is clinically relevant since (a) the rate of unintended childhood intoxication doubled between 2000 and 2013 (5–7), and (b) population follow-up of users with high-potency “skunk,” a high-quality strain of cannabis, indicates a 3- to 5-fold increase in psychotic episodes when self-administration commences at a reported age of less than 11 years (8). Therefore, understanding if pediatric exposure to THC (9, 10) causes lifelong impairments is of critical importance. Nevertheless, and while manifold data exist on adverse THC effects in utero (11–13) even with transgeneration consequences (14, 15), mechanistic insights in the cellular outcome of THC exposure during childhood and preadolescence remain fragmented (however, see refs. 16, 17).

Development of the mammalian brain is a protracted process; while gross neurogenesis ceases by 1–1.5 years of age in humans (18) (except the hippocampus; refs. 19, 20), the formation of synaptic contacts, their activity-dependent selection, pruning (refinement), and myelination become complete by late adolescence (i.e., 22–24 years of age in humans; refs. 21, 22). Particularly, the stabilization and selection of synaptic contacts that drive meaningful information in corticolimbic networks together with postnatal avalanches of programmed cell death that reduce neuronal redundancy (23, 24) can be sensitive to exposure to psychoactive drugs, such as THC (25). These notions are congruent with neuroanatomical changes (defined as gray matter thinning in, for example, amygdala; ref. 26) that appear as early as after the second exposure to cannabis in adolescents (26, 27). Most of the experimental work involving THC focused on neuronal contingents that express CB1 cannabinoid receptors (CB_1_Rs), including cholecystokinin^+^ (CCK^+^) interneurons (28) and pyramidal cells (12, 29, 30), with the rationale that THC can impair the precise sequel of neuronal migration and morphogenesis by occluding endocannabinoid signaling at CB_1_Rs (11, 31, 32). Existing knowledge on CB_1_R-dependent mechanisms is also critical in disease settings, since functional deficits in, for example, CCK^+^ interneurons, have been associated with neuropsychiatric disorders (33, 34) and epileptogenesis (35).

CB_1_Rs are the critical signal transduction components of the endocannabinoid system in neurons (31, 36) and usually operate as G_i_-coupled GPCRs at the cell surface (37, 38). Since endocannabinoids are arachidonic acid–derived eicosanoids (39), their lipophilicity suggests the engagement of cell surface and putative intracellular receptors. Therefore, a recent string of discoveries on intracellular CB_1_Rs, including those that putatively partition to mitochondria (40–43), posits that ligand engagement of such receptors at the mitochondrial outer membrane could directly impact a cell’s ability of oxidative phosphorylation (OXPHOS) and, ultimately, ATP production. Conspicuously, THC also exhibits a lipophilic character (44) and accumulates in lipid bilayers, changing their fluidity (45, 46). Thus, and besides binding to CB_1_Rs, THC can also alter key biophysical properties of cellular membranes (45, 47–49), which seems particularly relevant when developing neurons undergo a > 1000-fold membrane expansion to sustain the formation of their axons and dendrites. This concept, regardless of the particular subcellular positioning of CB_1_Rs, builds on the findings that both cannabis extracts and THC (at concentrations ≥ 400 nM; ref. 40) significantly reduce oxygen consumption in brain homogenates both in vitro and in vivo (50–52), suppress respiration by inhibiting mitochondrial electron transport (53), and induce mitochondrial swelling (54, 55). Thus, an inference can be made to THC also having a direct and detrimental impact on neuronal morphology and connectivity in a CB_1_R-independent manner. However, neither the precise cellular consequence nor any route of rescue of THC-induced mitochondrial failure in a developmental setting is known.

Here, we show that THC exposure of healthy mice during the early postnatal period induces significant cellular rearrangements in the hippocampus, which involve both CB_1_R^+^ and CB_1_R^–^ neuronal subclasses. We then used quantitative proteomics to demonstrate that long-lasting effects of THC exposure during the preadolescent period in mice impacts the expression of key molecular constituents of the mitochondrial respiratory chain. We then used the experimental power of high-throughput IncuCyte imaging to test the dose-dependence of THC effects on neuronal survival and neurite outgrowth, and to link these to the rapid elimination of the mitochondrial membrane potential (MMP) in THC-exposed neurons. By performing nanoindentation analysis, we demonstrate that THC at concentrations > 7.5 μM disrupts neuronal membrane stiffness (a mark of increased membrane fluidity) in a CB_1_R-independent fashion in vitro. Subsequently, we explored to what extent stabilizing intracellular pH, bypassing glycolysis, activating mitochondrial adenylyl cyclase (41) or the use of neuroprotective compounds that target the mitochondrial voltage-dependent anion channel (VDAC) could counteract adverse THC effects. Cumulatively, our data define key sites and mechanisms of neuronal vulnerability to THC and offer prototypic strategies of rescue, at least in vitro.

## Results

### THC binds to CB_1_Rs and induces neuronal activity in juvenile mice.

In utero exposure to THC or synthetic CB_1_R agonists (56) selectively reduces the number, morphological complexity, and local innervation of CCK^+^/CB_1_R^+^ interneurons (12, 57) in the fetal hippocampus (with trends for parvalbumin [*Pvalb*], somatostatin [*Sst*], and other subclasses; ref. 12) and leaves adaptive neuronal plasticity permanently reduced in affected offspring (11). However, whether this sensitivity persists during postnatal development (that is, until adolescence) remains unknown. This question is particularly relevant considering that many interneurons (including the CCK^+^/CB_1_R^+^ subclass) migrate, morphologically differentiate, and establish their target-selective innervation pattern during the first postnatal weeks in rodents (58, 59). In addition, many neuronal subclasses, including principal cells, express CB_1_Rs at moderate levels throughout life (30, 56, 60, 61).

To define the developmental dynamics and ligand competence of CB_1_Rs during corticolimbic development, we undertook saturation binding experiments with either [^3^H]CP55,940 (Supplemental Figure 1A; supplemental material available online with this article; https://doi.org/10.1172/jci.insight.135418DS1) or [^3^H]SR141716A (62) (Supplemental Figure 1B). The total number of binding sites (B_max_) for [^3^H]CP55,940 (expressed as fmol/mg protein throughout this article) increased significantly along successive developmental stages (356.4 ± 222.4 [E18.5]/19% of adult, 145.0 ± 123.0 [P2]/12% of adult, 623.2 ± 11.9 [P16]/51% of adult and 1214.2 ± 136.4 [adult]; Supplemental Figure 1A) without the K_d_ being altered. [^3^H]SR141716A binding followed the same trend for B_max_ (215.9 ± 17.9 [E18.5]/18% of adult, 371.4 ± 237.5 [P2]/19% of adult, 1079.9 ± 476.1 [P16]/57% of adult, and 1909.7 ± 542.6 [adult]; Supplemental Figure 1B) with no consistent effect on K_d_. We also tested if incrementing concentrations of plant-derived THC (pTHC; 10, 30, 100, and 300 nM) displace [^3^H]CP55,940 (0.5 nM): the half-maximal inhibitory concentration for pTHC (IC_50_) remained unchanged throughout (37 ± 9 nM [E18.5], 25 ± 4 nM [P2], 28 ± 8 nM [P16], and 25 ± 2 nM [adult]; *P* > 0.3; Supplemental Figure 1C). These data show that CB_1_R binding is developmentally regulated and suggest that the efficacy of pTHC at binding CB_1_Rs (and hence potentially biasing CB_1_R-mediated signaling) is not affected by structural modifications or differential receptor pharmacology during postnatal development in mouse.

**Figure 1:**
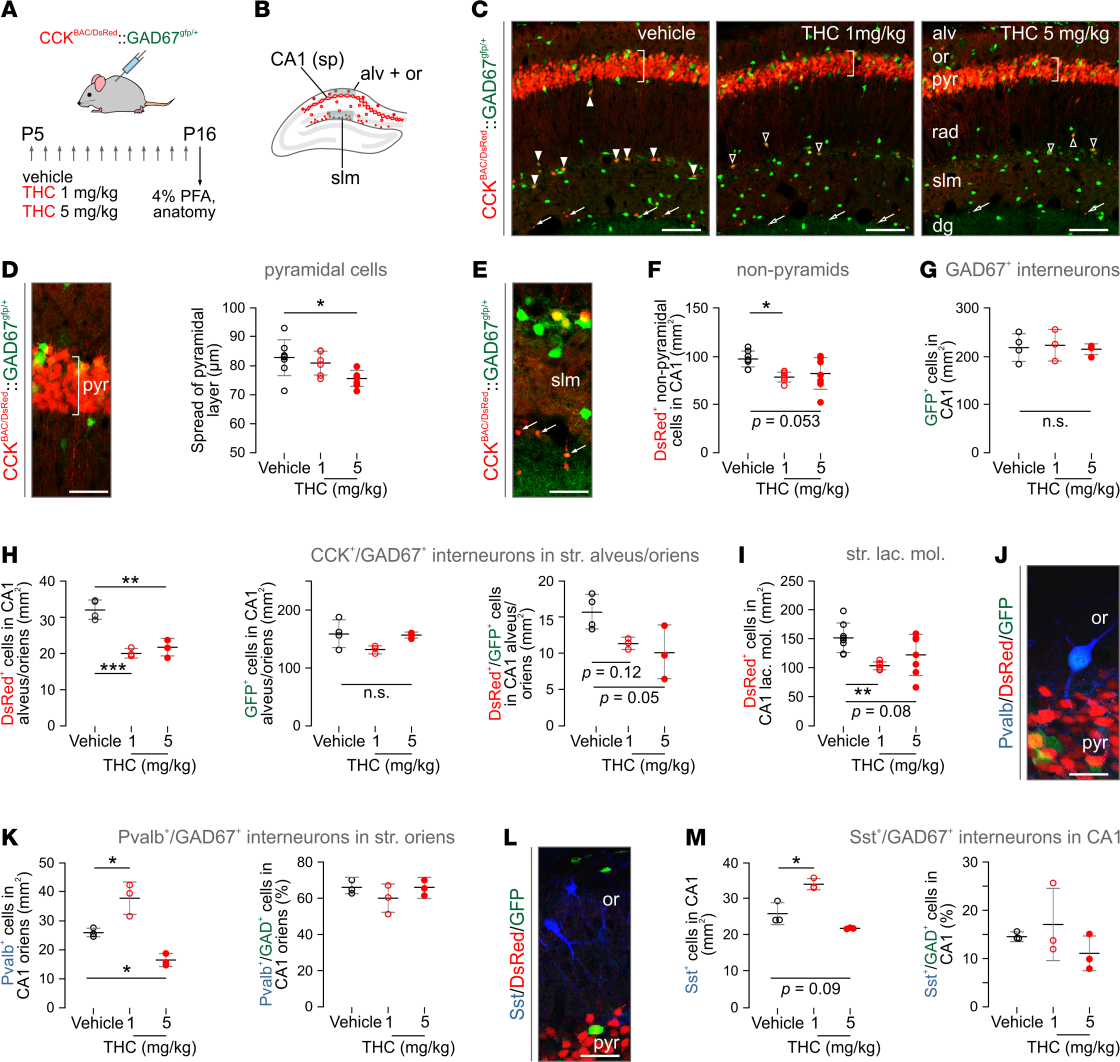
THC exposure during P5–P16 induces neurochemical deficits in CA1 hippocampal neurons. (**A**) Experimental paradigm in CCK^BAC/DsRed^ and *Cck*^BAC/DsRed^*Gad1*^gfp/+^ mice. Daily injections had an average volume of 100 μL, but final volume was adjusted to the individual body weight; *n* = 3–8 mice/genotype/treatment (*n* = 3–4 mice/treatment for *Cck*^BAC/DsRed^*Gad1*^gfp/+^ line). (**B**) Schematic outline of the dorsal hippocampus, with red circles denoting the localization of DsRed^+^ neurons. (**C**) Representative images from DsRed^+^/GFP^+^ hippocampi after vehicle or THC treatment. Vertical bar over the pyramidal layer shows the general approach to measure cell spread within. Solid arrowheads point to DsRed^+^/GFP^+^ interneurons in control, whereas open arrowheads denote residual cells upon THC exposure. Arrows point to small-diameter DsRed^+^ neurons at the deep stratum lacunosum moleculare (slm; see also Supplemental Figure 3A). (**D**) High-resolution image of pyramidal cells in hippocampal CA1, with vertical bar illustrating a vector to measure cell spread (left) with quantitative data (right). (**E**) DsRed^+^/GFP^+^ neurons in slm. Arrows point to small-diameter DsRed^+^ signal. (**F** and **G**) The density of DsRed^+^ (**F**) but not GFP^+^ neurons (**G**) significantly decreased in nonpyramidal layers of the CA1 subfield (qualifying as interneurons by location) after THC treatment. (**H**) Likewise, the density of DsRed^+^/GFP^+^ interneurons in strata alveus/oriens (but not of the GFP^+^ neuronal contingent) became significantly reduced upon THC treatment. (**I**) Similar changes were seen in stratum lacunosum moleculare. (**J**) Representative photomicrograph showing the distribution of Pvalb^+^/GFP^+^ interneurons in nonpyramidal CA1. (**K**) THC-induced dose-dependent changes in Pvalb^+^ interneuron density in stratum oriens. Note that THC treatment did not affect the probability of Pvalb and GFP colocalization. (**L**) Histochemical detection of Sst^+^ interneurons in the hippocampus. (**M**) THC induced dose-dependent changes in the density of but not the probability of colocalization with GFP for Sst^+^ interneurons. Cell counts were normalized to a surface area of 1 mm^2^. Data were expressed as mean ± SD; *P < 0.05, **P < 0.01, ***P < 0.001 (versus control; 1-way ANOVA followed by Bonferroni’s post hoc test). Scale bars: 120 μm (**C**), 25 μm (**D** and **E**), 10 μm (**J** and **L**). str., striatum; alv, str. alveus; dg, dentate gyrus; or, str. oriens; PFA, paraformaldehyde; pyr, str. pyramidale; rad, str. radiatum.

Next, we asked if pTHC can affect neurons in juvenile mice by using c-Fos activation as a readout (63, 64). In P9 mice, pTHC (5 mg/kg) induced c-Fos expression in the central amygdaloid nucleus (0.2 ± 0.2 [vehicle] versus 22 ± 2.7 [pTHC] cells/section; *P* < 0.01) 2 hours after its s.c*.* drug administration (Supplemental Figure 1, D and E). These data are consistent in magnitude and regional specificity with earlier observations in adult (63, 64) and demonstrate that systemic bolus injections of pTHC, particularly in a repeated administration regime (Figure 1A), at doses similar to those tested here could affect the postnatal development of the corticolimbic circuitry.

### Neuronal subtype sensitivity to THC in the juvenile hippocampus.

We have taken advantage of *Cck*^BAC/DsRed^ reporter mice (65), which reliably tag both pyramidal cells and interneurons (66), to determine pTHC effects (1 or 5 mg/kg/day during P5–P16) in the CA1 subfield of the hippocampus (Figure 1B). Thinning of the CA1 pyramidal cell layer (i.e., a reduced distance between the uppermost and lowermost pyramidal cell at P16) was significant in mice exposed to 5 mg/kg (82.7 ± 8.1 μm [vehicle] versus 75.6 ± 2.8 μm [pTHC]) but not 1 mg/kg pTHC (80.9 ± 4.2 μm; Figure 1, C and D and Supplemental Figure 2, A–C). Next, we found that the density of DsRed^+^ nonpyramidal cells (putative interneurons in any other cell layer; Figure 1E) was also reduced in pTHC-exposed mice (97.2 ± 8.7 [vehicle] versus 78.19 ± 5.7 [1 mg/kg], 82.47 ± 17.4 cells/mm^2^ [5 mg/kg], *P* = 0.016 and *P* = 0.053, respectively; Figure 1F), which were particularly pronounced in the oriens and molecular layers (*P* < 0.01; Figure 1, H and I) but not radiatum (Supplemental Figure 2, D–F). These data show that pTHC is adverse to hippocampal development in juvenile Cck^BAC/DsRed^ mice by either limiting the pool size of neuronal progenies or disrupting *Cck* gene transcription.

We then used a dual-transgenic CCK^BAC/DsRed^GAD67^gfp/+^ reporter line in which interneurons are either DsRed^+^/GFP^+^ or GFP^+^ alone. The number of GFP^+^ interneurons did not change upon pTHC treatment in either the total CA1 field (219.1 ± 28.6 [vehicle] versus 223.4 ± 32.9 [1 mg/kg pTHC], 215.0 ± 10.3 cells/mm^2^ [5 mg/kg pTHC], *P* > 0.8; Figure 1G) or its sublayers (Figure 1H and Supplemental Figure 2, E and F) leaving a significant change in the normalized percentage of DsRed^+^/GFP^+^ dual-labeled interneurons (Figure 1, H and I). We interpret these findings as *Cck* gene regulation rather than interneuron survival, per se, being adversely affected by pTHC in the juvenile brain — at least at doses similar to those tested herein. Moreover, and since we do not find marked apoptosis of hippocampal neurons 24 hours after the last pTHC injection (Supplemental Figure 3, A–F), we hypothesize that cell death, if any, occurs instead rapidly after initiating pTHC treatment.

Next, we reasoned that impaired neuronal development might not be limited to CB_1_R^+^ neuronal contingents. Therefore, we first determined the density of *Pvalb*^+^ interneurons, which lack CB_1_Rs (67), and found it significantly increased upon 1 mg/kg THC administration. Conversely, 5 mg/kg THC reduced their number (Figure 1, J and K). Likewise, the density of *Sst*^+^ interneurons was increased upon exposure to 1 mg/kg THC, with no significant alteration reported at 5 mg/kg (Figure 1, L and M). Neither THC dose altered the probability of either *Pvalb*^+^/GFP^+^ or *Sst*^+^/GFP^+^ colocalization (Figure 1, K and M). These data show that CB_1_R^–^ interneurons (for Sst, see ref. 68) are also affected by THC, which is compatible with earlier data on *Pvalb* deregulation in constitutive CB_1_R-KO (*Cnr1*^–/–^) mice (69) and suggests broad developmental THC effects.

### Long-lasting proteome modifications upon pTHC exposure of the preadolescent brain.

**Figure 2:**
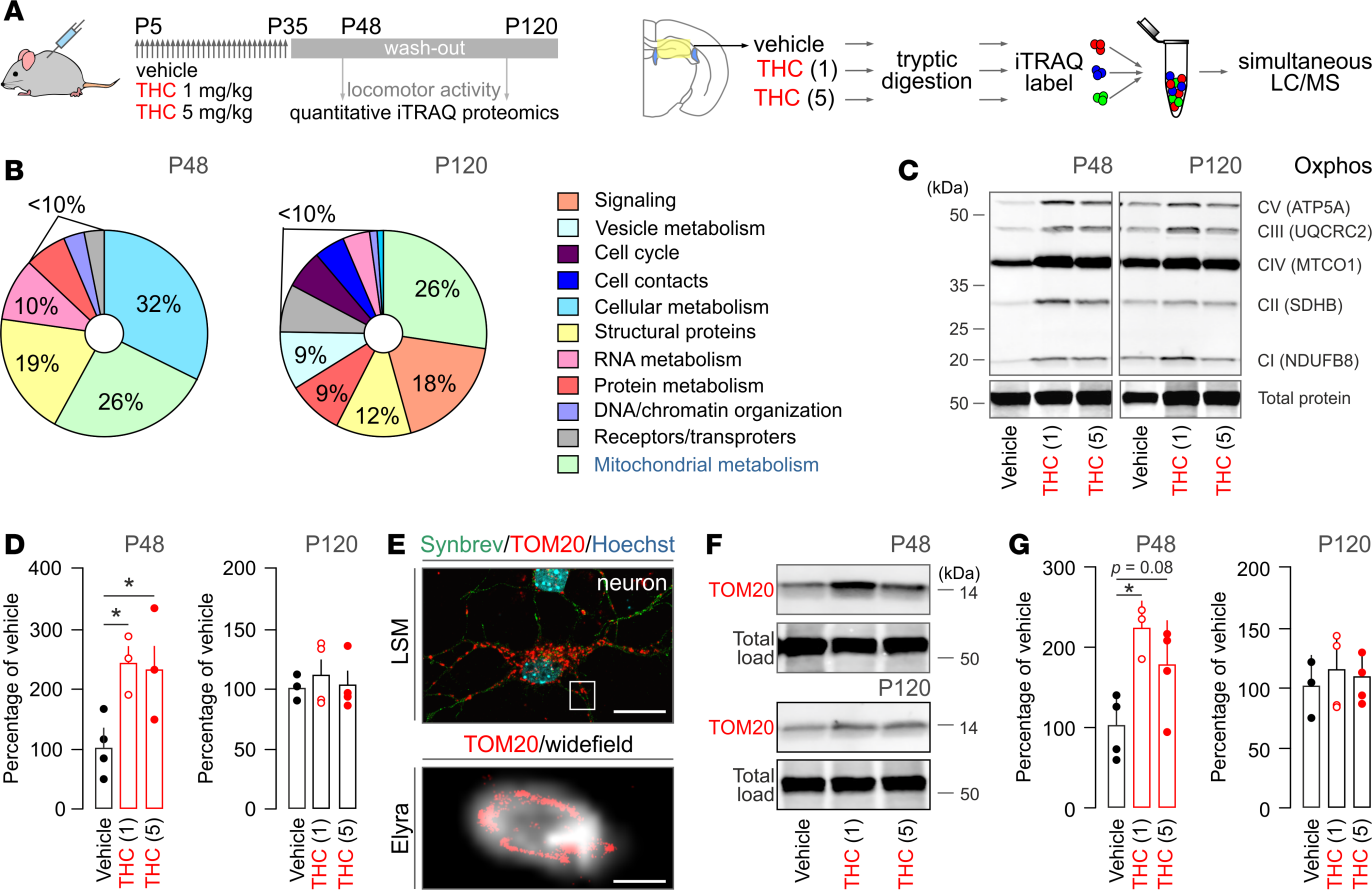
Long-lasting alterations in the mouse hippocampal proteome upon THC exposure identify a mitochondrial site of vulnerability. (**A**) Schematic illustration of the analysis pipeline. THC and vehicle were administered daily during the period of P5–P35. Tissue collection was on either P48 or P120 (*n* = 5 fetuses/group/time point from independent pregnancies) followed by iTRAQ proteomics. (**B**) Graphical illustration of the functional assignment of protein targets to gene ontology (GO; https://www.uniprot.org) clusters on P48 or P120. (**C**) Representative Western blot colabeled for molecular constituents of the 5 mitochondrial respiratory chain complexes on P48 and P120 (such as NADH dehydrogenase [ubiquinone] 1 β subcomplex subunit 8 (NDUFB8; complex I [CI]); succinate dehydrogenase [ubiquinone] iron-sulfur subunit [SDHB; CI]); cytochrome b-c1 complex subunit 2 [UQCRC2; CIII]; cytochrome c oxidase subunit 1 [MTCO1; CIV]; and ATP synthase subunit α [ATP5A; CV]). Cy5 dye labeling was used to normalize protein load (Supplemental Figure 5). (**D**) Cumulative Western blot results on the levels of the mitochondrial oxidative phosphorylation (OXPHOS) machinery. (**E**) TOM20 immunoreactivity in cortical neurons detected by laser-scanning microscopy (upper). Synaptobrevin (Synbrev) was used as a presynaptic/axonal marker. Subsequently, super-resolution microscopy (Zeiss ELYRA) confirmed the localization of TOM20 in mitochondria (lower). (**F**) Representative Western blots labeled for TOM20 (or total protein load; Supplemental Figure 5) at P48 or P120. (**G**) Quantitative data are from *n* ≥ 3 animals/group. Data were expressed as mean ± SEM; *P < 0.05 (versus control; 1-way ANOVA followed by Bonferroni’s post hoc test). Scale bars: 20 μm (**E**, upper), 500 nm (**E**, lower).

In the fetal brain, iTRAQ proteomics revealed that THC dysregulates 33 proteins, most of which are involved in neurite outgrowth by priming protein synthesis, cytoskeletal modifications, and cell adhesion (11). Here, we hypothesized that pTHC could modulate alternative sets of proteins when neurons are beyond the completion of their primary developmental programs and that these sets of proteins are required for neuronal subclass specification instead. We have favored a broad-scale unbiased proteomics approach (using 8-plex iTRAQ; ref. 11) after completing an experimental paradigm that included daily injections of 1 or 5 mg/kg pTHC during the period of P5–P35 (i.e., the entire preadolescent period in mouse) and washout for either 14 (P48) or 85 days (P120; Figure 2A). Our choice of these washout periods was 2-fold: to ensure (a) that bioactive THC metabolites are no longer present (70) with complete clearance reported at > 72 hours upon chronic administration (71–73) and (b) that biologically meaningful and persistently altered targets are captured. We monitored spontaneous exploratory and anxiety-like behaviors (a parameter of the cannabinoid tetrad) to confirm neither acute THC-induced hypomotility nor anxiety (Supplemental Figure 4).

Among the 31 proteins (PRIDE accession no. PXD010802) significantly different at P48, 25 and 3 proteins were dose-dependently increased and decreased, respectively (Figure 2B). Three proteins were significantly increased without a consensus effect. A particularly large cluster of proteins (8 of 31) were identified as relevant to mitochondrial function according to their gene ontology (GO) classification (Table 1). Other entries belonged to cellular processes of highest energy demand (cytoskeletal rearrangement, RNA turnover, chromatin modifications). Given the significant upregulation of respiratory-chain (*Atp5h*, *Atp6v1e1*), antioxidant (*Prdx1/2*, *Sod2*), and ATP synthesis–related proteins (*Nme2*), we posited that pTHC could be adverse for neuronal survival and function determination in the juvenile brain by disrupting neuronal (or glial) bioenergetics. Accordingly, increased protein abundance was taken to be indicative of adaptive modifications to counter pTHC action. This concept is compatible with recent data on the disruption of complex I of the mitochondrial respiratory chain (40, 41, 74), irrespective of the subcellular positioning of CB_1_Rs on the plasmalemma, mitochondrial membranes, or both (75, 76). Another significant finding is the reduced availability of the mitochondrial transfer RNA (tRNA) nucleotidyl transferase (*Trnt1*) whose disruption in genome-wide association studies (GWAS) is associated with cerebellar developmental delay (77–79), ataxia, and reduced cellular respiration (80).

THC effects in the adult brain are thought to be transient. This hypothesis is unlikely to apply to developing cellular systems because the imprinting of particular errors during short episodes of organogenesis is carried forward in a cascade of adverse cellular events (81). Therefore, we tested if proteome modifications persist in P120 offspring (Figure 2B). Indeed, the levels of 186 proteins changed significantly, with dose-dependent increase (*n* = 162) and decrease (*n* = 10) seen most commonly (Table 1). Despite many proteins assigned to cellular signaling (*n* = 34), synaptic vesicle turnover (*n* = 17), receptors (*n* = 14), and transporters (*n* = 11), 49 proteins were classified as participating in mitochondrial function (26%; Table 1). Among the significantly upregulated proteins, NADH-ubiquinone oxidoreductase subunits (*Ndufa6*, *Ndufa13*, *Ndufs6*, *Ndufs3*), ATP synthase subunits (*Atp5h*, *Atp6v1e1*, *Atad3*, *Afg3l2*), and mitochondrial VDAC subunits (*Vdac1–3*) were prevalent, and a total of 13 NADH dehydrogenase subunits (complex I), 3 cytochrome b–c1 complex subunits (complex III), and 17 proteins implicated in ATP synthesis (ATP synthase subunits, coupling factors, translocases) were significantly affected as well. In contrast, catalase (*Cat*), mitochondrial carnitine O-palmitoyltransferase (*Cpt2*), and dimethylarginine dimethylaminohydrolase 2 (*Ddah1*) were reduced, further supporting the persistent maladaptation of ATP synthesis, free radical defense, and NO signaling. Comparative analysis of P48 and P120 samples revealed the permanent deregulation of α-enolase (*Eno1*), mitochondrial ATP synthase subunit d (*Atp5h*), and V-type proton ATPase subunit E1 (*Atp6v1e1*) in mitochondria. Thus, there is reason to believe that exposure of the juvenile brain to repeated doses of THC similar to those tested here imposes lifelong modifications to cellular bioenergetics, at least in rodents.

Next, we validated the upregulation of mitochondrial complexes I, III, and V subunits and auxiliary proteins by simultaneously detecting components for each of the 5 respiratory complexes of the mitochondrial OXPHOS machinery that are different in molecular weight (Figure 2C and Supplemental Figure 5). Both 1 and 5 mg/kg THC significantly increased the abundance of OXPHOS subunits at P48 (*P* < 0.05; *n* > 3/group; Figure 2D). However, significant differences were not resolved in P120 samples when normalizing OXPHOS readouts to the total protein load (Figure 2, C and D).

The translocase of outer mitochondrial membrane 20 (*Tom20*) is central to the recognition and translocation of proteins that are synthesized in the cytosol and destined to mitochondria (82). Superresolution microscopy showed that TOM20 indeed resides in the inner membrane of neuronal mitochondria (Figure 2E). Given that translocase activity gates mitochondrial integrity, we have probed if pTHC also affects TOM20 protein levels. At P48 but not P120 (Figure 2F), TOM20 was significantly enriched in pTHC-exposed hippocampi (Figure 2G and Supplemental Figure 5). Overall, these data show that pTHC exposure of the juvenile hippocampus within the dose range tested here imparts long-lasting molecular maladaptation of cellular bioenergetics, and they outline a critical target for pharmacological rescue.

### CB1R-dependent and -independent mechanisms of neuronal growth arrest.

**Figure 3:**
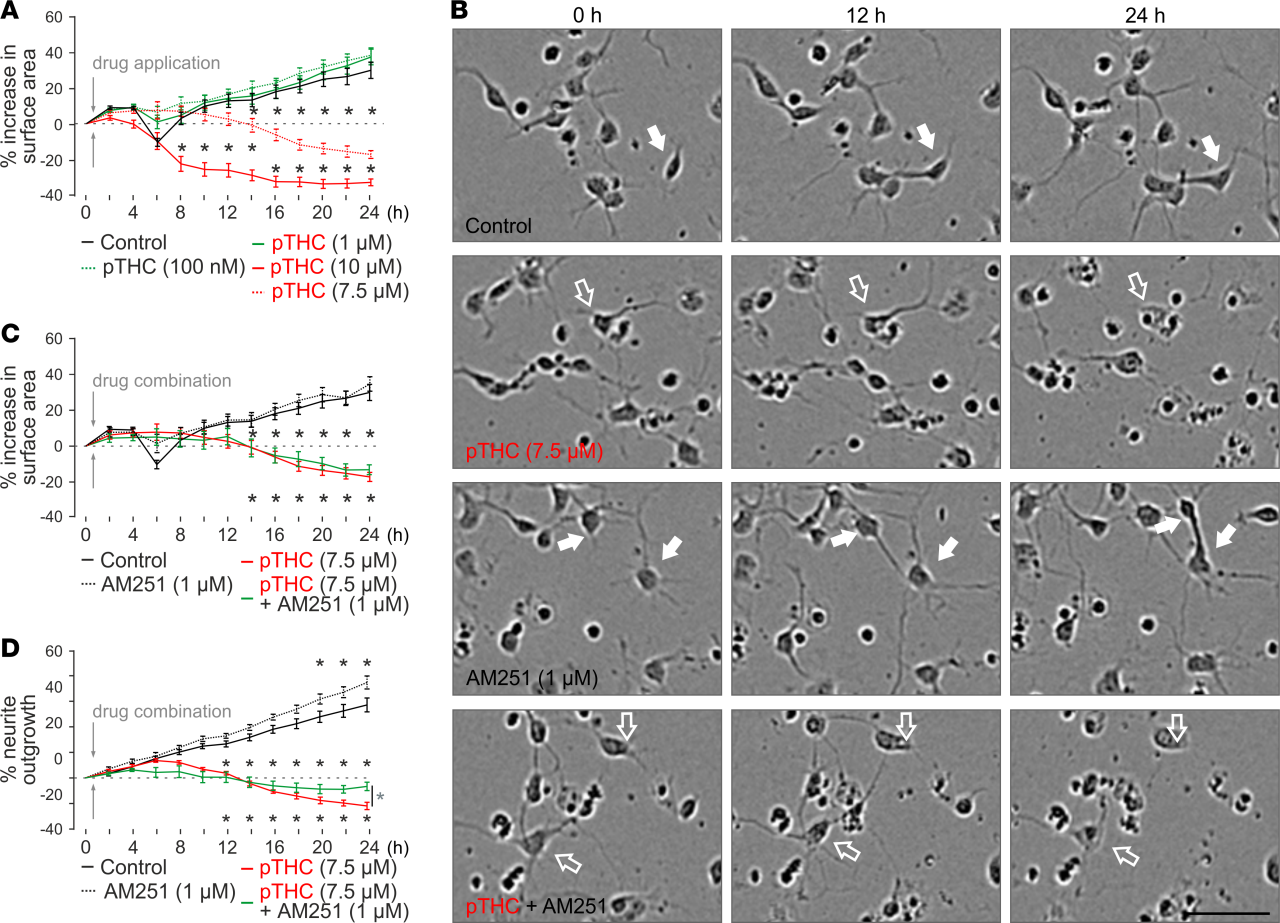
High-throughput time-lapse analysis of THC-induced growth retardation of cortical neurons in vitro. (**A**) Plant-derived THC (pTHC) dose-dependently reduced the surface area occupied by neuronal somata, with 10 μM THC inducing significant cell death. (**B**) Representative phase-contrast images of cultured cortical neurons exposed to the drugs indicated. Solid and open arrows point to live and fragmented neurons, respectively. A THC concentration of 7.5 μM was used that showed a slow and protracted effect, amenable to pharmacological modulation. Scale bar: 50 μm. (**C**) At 7.5 μM THC concentration, AM251 was ineffective to rescue neuronal survival, as inferred from cell surface area. (**D**) However, AM251 induced significant recovery of neurite outgrowth at 24 hours (Supplemental Figure 6). Data were collected and analyzed by using an IncuCyte Zoom imaging platform with a loop time of 2 hours. *P < 0.05 (versus control [black] or THC + AM251 [in **D**, green]; 2-way ANOVA with Bonferroni’s post hoc correction). Data were expressed as mean ± SEM with *n* = 8–18 technical replicates each.

In developmental models of THC toxicity, reduced neurite outgrowth and dose-dependent shrinkage of the perikarya are taken as key parameters (83). Here, we have used high-throughput IncuCyte live-cell imaging (84) to differentiate CB_1_R-dependent and -independent components of neuronal growth upon THC application in vitro; pTHC dose-dependently reduced the surface area occupied by neuronal perikarya over 24 hours (Figure 3, A and B) with THC concentrations > 7.5 μM rapidly and irreversibly compromising neuronal survival (at 24 hours, 38.0% ± 4.1% growth [100 nM], 37.1% ± 4.2% growth [1 μM], –17.1% ± 2.0% loss [7.5 μM], and –32.6% ± 1.9% loss [10 μM]). AM251 (1 μM), an inverse CB_1_R agonist considered to be cell permeant (40, 85), was ineffective in rescuing neuronal survival, as inferred from cell surface area (Figure 3C). In contrast, AM251 significantly reduced the loss of neurite outgrowth by 24 hours, which was evident for both 7.5 μM (Figure 3D) and 10 μM THC (Supplemental Figure 6). These data suggest that retaining neuronal integrity might have a significant CB_1_R-independent component (at around a concentration of 7.5 μM), while retarded neurite outgrowth is a CB_1_R-dependent process (31, 60).

### THC disrupts mitochondrial integrity in developing neurons.

**Figure 4:**
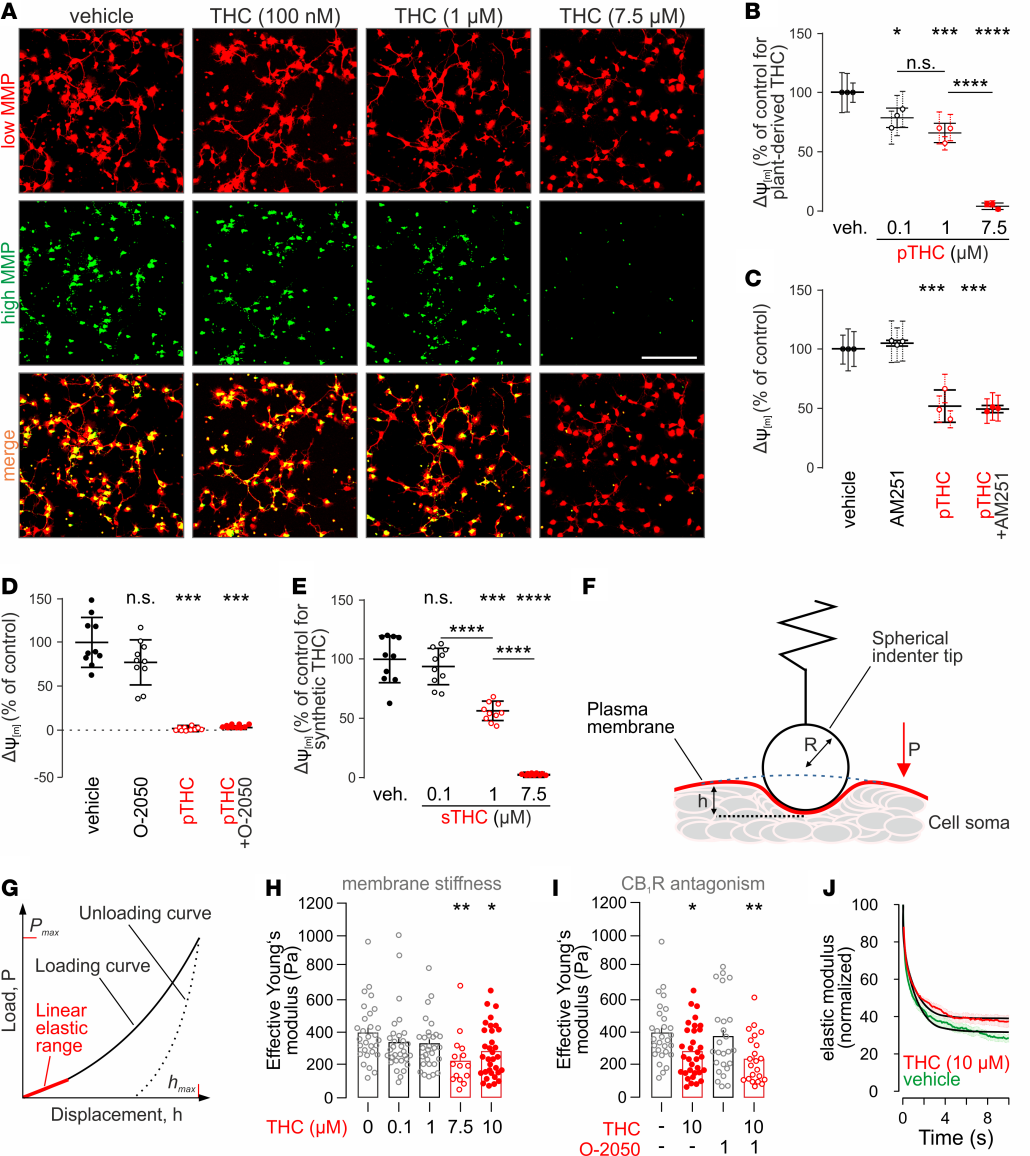
THC disrupts the mitochondrial membrane potential and changes biophysical properties of the neuronal plasma membrane in vitro. (**A**) Representative images of cortical primary neurons exposed to vehicle or to the THC concentrations indicated and processed by the Mito-ID assay. Note that THC-induced mitochondrial damage reduced the high mitochondrial membrane potential (MMP), while leaving its low component unchanged. Scale bar*:* 100 μm. (**B**) Quantitative analysis of the high/low MMP ratio revealed dose-dependent THC effects. (**C** and **D**) MMP in the presence of pTHC with or without AM251 (1 μM; **C**) or O-2050 (100 nM; **D**), both cell-permeable CB_1_R antagonists (40, 85). (**E**) Synthetic THC (sTHC) was as efficacious in disrupting the MMP as pTHC. Dose-response relationship is shown. (**F**) Illustration of the cell indentation procedure including relevant parameters for the calculations of the effective Young’s modulus: P, load induced by indenter tip; h, displacement; R, indenter radius. (**G**) Load displacement curve. The linear elastic response of the loading curve (red) was used to calculate cellular surface stiffness following the Hertz model (141, 142). (**H** and **I**) The effective Young’s modulus was dose-dependently reduced by THC (**H**), with O-2050 unable to prevent a significant reduction in membrane stiffness brought about by 10 μM THC (**I**). (**J**) Stress-relaxation curves indicate the altered viscoelastic profile of THC-exposed neurons relative to controls. Data in **B**–**E** were normalized to control. Data in **B** and **C** were expressed as mean ± SD of triplicates with individual experiments (circles) using *n* = 10 replicates each. Data in **D** and **E** were expressed as the mean ± SD of 10 parallel observations. Nanoindentation data were on *n* = 15–33 cells/group and expressed as mean ± SEM. *P < 0.05, **P < 0.005, ***P < 0.001, ****P < 0.0001; 1-way ANOVA followed by Bonferroni’s post hoc test was used for MMP measurements and nanoindentation data.

The above growth delay in cultured neurons in conjunction with our molecular target discovery by proteomics suggest the THC-induced disruption of mitochondrial integrity and function. Here, we devised a neuronal model in vitro to interrogate mitochondrial integrity based on the existence (or lack) of the MMP (or Δѱ_m_; ref. 86), a critical component of the mitochondrial proton driving force (Δp), which regulates the phosphorylation of ADP into ATP (Supplemental Figure 7). By using the MITO-ID assay (Figure 4A), we found that pTHC dose-dependently lowers the MMP, reaching significance at concentrations ≥ 100 nM (Figure 4, A and B). At a pTHC concentration of 7.5 μM, the MMP was abolished completely (*P* < 0.001; Figure 4B) within 30 minutes. Considering that the loss of MMP is directly linked to early stages of cell death, these findings are compatible with the THC-induced loss of neuronal survival, as inferred from cell surface area (Figure 3A). Next, we tested if either AM251 (1 μM) or O-2050, another CB_1_R antagonist, was able to prevent pTHC effects. Neither drug showed beneficial effects on MMP at any concentration tested (>100 nM; note that Figure 4, C and D, show 1 μM and 7.5 μM THC, respectively). Finally, we used HEK293 cells that do not express CB_1_Rs to confirm that MMP disruption by THC is a CB_1_R-independent process (Supplemental Figure 8). These data are in concordance with earlier observations that cell surface CB_1_Rs are unlikely to control mitochondrial bioenergetics (87–89).

### Both synthetic THC and pTHC induce neuronal injury.

THC preparations extracted from *Cannabis spp*. inherently contain minute amounts of other phytocannabinoids, terpenes, and flavonoids. Therefore, one might argue that any biologically active contaminant could influence (or even account for) pTHC effects. We have addressed this hypothesis by using synthetic THC (sTHC; >99% sourced from 2 vendors) at equimolar concentrations (Figure 4E and Supplemental Figure 9). The IC_50_ of sTHC was equivalent to pTHC in radioligand binding experiments (Supplemental Figure 9A) and lacked toxicity at 1 μM concentration (Supplemental Figure 9B), while reducing neuronal growth (Supplemental Figure 9C) and disrupting MMP with a dose-effect relationship equivalent to that of pTHC (Figure 4E and Supplemental Figure 9D). In sum, many aspects of pTHC versus sTHC toxicity on developing neurons are comparable, yet high sTHC concentrations (10 μM) seem less detrimental for neuronal survival than those of pTHC. This difference might be attributed to the cooperative bioactivity of residual plant molecules (90, 91).

### Catastrophic membrane failure upon acute THC exposure in vitro.

By being a lipophilic compound, THC can change membrane fluidity (45, 46). Assuming that THC effects are indiscriminate in vivo and exhibit a significant CB_1_R-independent component in vitro, we sought to address if THC reduces the stiffness of the neuronal plasma membrane (that is, increases its fluidity). To this end, we have combined nanoindentation (Figure 4F) and CB_1_R pharmacology, with an indentation depth of 1 μm chosen to limit access to the plasmalemma (92) without biasing the measurements by the resistance of membrane-associated deeper cytoplasmic structures. Nanoindentation measurements of the effective Young’s modulus (Figure 4G) revealed that the stiffness, as a measure of membrane fluidity, of the neuronal plasmalemma is dose-dependently reduced by pTHC, reaching statistical significance at 7.5 μM (55% of control, *n* = 15; 10 μM, 70.5% of control, *n* = 33). Vehicle-treated neurons showed a Young’s modulus of 395.2 ± 33.9 Pa (*n* = 29; Figure 4H). O-2050 (1 μM) failed to prevent pTHC effects, emphasizing their CB_1_R-independent nature (Figure 4I). Last, THC also altered the viscoelastic modulus of neuronal membranes (Figure 4J), which is a measure of cellular rebound upon constant indentation. Our data corroborate previous reports on the effective Young’s modulus in cultured embryonic cortical neurons measured with atomic force microscopy (93), and they underscore that high THC concentrations interfere with the physical stability of neuronal membranes.

### Targeting soluble adenylyl cyclase can mitigate pTHC effects.

**Figure 5:**
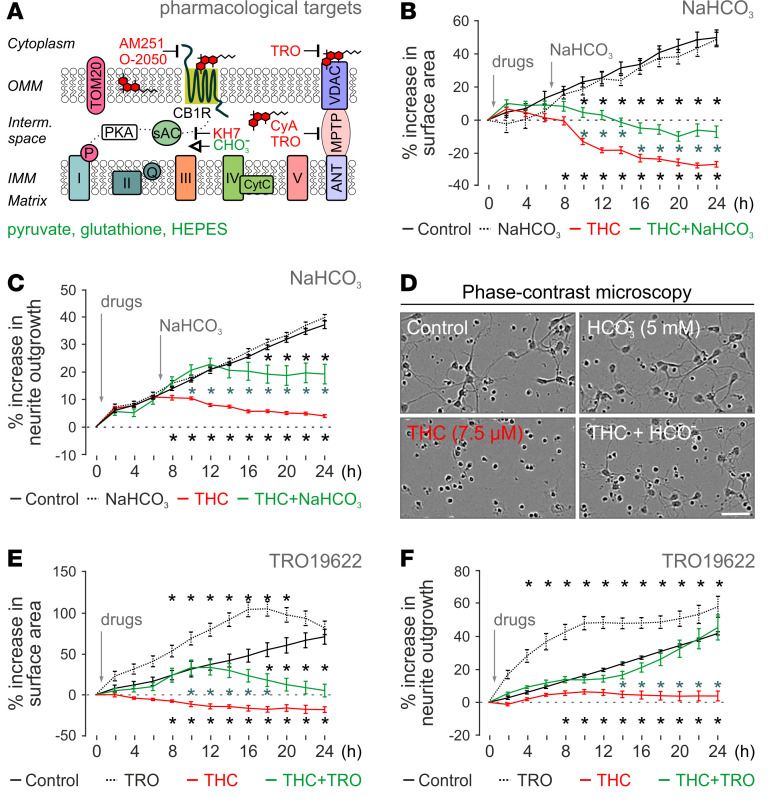
Rescue of THC-induced neuronal impairment by improved or bypassed mitochondrial function. (**A**) Schematic outline of drug action on the mitochondrial electron transport chain. CyA, cyclosporine A; TRO, TR019622. (**B** and **C**) Beneficial effects of 5 mM NaHCO_3_ on THC-induced (7.5 μM) neuronal death (**B**) and slowed neurite outgrowth (**C**). (**D**) Representative phase-contrast (PC) images 24 hours after THC exposure with/without NaHCO_3_. Scale bar*:* 25 μM. (**E** and **F**) Time-resolved effects of TR019622 on THC-induced growth retardation, including cell survival (**E**) and neurite outgrowth (**F**). Data in **B**–**F** were produced by high-throughput live-cell imaging (IncuCyte) with *n* ≥ 8 replicates and expressed as mean ± SEM. Asterisks in black denote *P* < 0.05 versus control, while asterisks in blue correspond to *P* < 0.05 versus THC (2-way ANOVA followed by Bonferroni’s post hoc correction).

THC is known to reduce cellular respiration by inhibiting complexes I–III of the electron transport chain (40, 41, 87). Soluble adenylyl cyclase (sAC) in the mitochondrial intermembrane space can convert ATP to cAMP, whose feedback stimulates OXPHOS and, thus, ATP production (94–96). Bicarbonate (HCO_3_^–^) activates SAC, whereas KH7 inhibits the enzyme (Figure 5A). As such, HCO_3_^–^ was successfully used to counteract the effects of THC on mitochondrial respiration in adult brain (41). Here, we find HCO_3_^–^ (5 mM) to significantly rescue pTHC-induced neuronal death at 24 hours (Figure 5, B–D, and Table 2). Moreover, HCO_3_^–^ increased neurite outgrowth (Figure 5, C and D) — yet without rescuing MMP (data not shown). Next, we used KH7 (5 μM), which failed to antagonize THC effects on both parameters tested (Table 2).

### Targeting VDAC ameliorates pTHC effects on both neuronal development and MMP.

VDACs are key molecular hubs that control the passage of small metabolites, cations (particularly Ca^2+^), and anions across the outer mitochondrial membrane (97). All 3 VDAC isoforms were upregulated by early postnatal pTHC exposure in vivo. These findings, together with the known interaction of VDAC with lipids and phytocannabinoids (98, 99), identify VDACs as a potential site of antagonism of detrimental pTHC effects. TR019622 (olesoxime) is a potent neuroprotective and neurotrophic compound targeting the mitochondrial VDAC and presumably preserving mitochondrial membrane integrity upon cellular injury (100, 101). Here, pretreatment of primary neurons with TR019622 not only attenuated the negative impact of pTHC on the survival and neurite formation of developing neurons (Figure 5, E and F, and Table 2), but also rescued THC-induced MMP collapse, at least at a THC concentration of 1 μM (56.33% ± 2.56% of control [pTHC] versus 74.37% ± 0.70% of control [pTHC + TR019622], *P* < 0.0001).

VDAC is part of the mitochondrial permeability transition pore (MPTP). Cyclosporin A prevents the opening and formation of the MPTP (Figure 5A). This accounts for cyclosporine A toxicity at concentrations > 1 μM (data not shown). When applying 100 nM cyclosporine A, we found a significant increase in neurite outgrowth, without a positive effect on either cell survival (Table 2) or MPP (data not shown). Nevertheless, we caution that cyclosporine A cytotoxicity is likely a confound in these experiments.

Last, we have tested if preventing cellular acidosis by HEPES, bypassing energy-consuming glycolysis by pyruvate supplementation, or using the antioxidant glutathione could counteract pTHC toxicity. Indeed, all 3 treatments yielded substantial positive outcome (Table 2), with glutathione being the most efficacious. Considering that these approaches critically stabilize mitochondrial function (HEPES, glutathione) or relieve glycose breakdown (and thereby a critical cellular strain on mitochondria), they complement and rationalize our findings on VDAC engagement as a means to alleviate pTHC effects.

## Discussion

Here, we show that repeated exposure of preadolescent healthy mice to THC at doses that have relevance to human recreational use (102) induce neuronal reorganization in the hippocampus; according to the US Food and Drug Administration (FDA), an experimental dose of 1 mg/kg THC for a mouse is equivalent to a dose of 0.081 mg/kg THC for humans, due to the different metabolic rates of the species. This supports the rationale of our in vivo experiments together with reports on childhood intoxications that indicate high substance concentrations (103, 104). Equally alarming is that THC concentrations can reach > 300 ng/mL in the milk of breastfeeding mothers and be detectable for > 6 days after the last exposure (105), which could see significant THC buildup in infants of small body weight. In this context, the endpoints of our short-term (P16) and long-term (P35) THC exposures can be seen as equivalent to 6 and 13.5 years, respectively.

Even though an expanding list of studies has tested THC effects on developing neurons, particularly in prenatal settings (11–13), it remains contentious if THC induces excess apoptosis or instead downregulates key neuronal identity marks (e.g., neuropeptides, neurotransmitters, receptors). Through the use of a dual transgenic reporter line, we show that it is more likely that THC limits neuronal specification by reducing the expression of neuronal subtype–selective marks than by inducing indiscriminate cell death. Moreover, we extend data beyond neuronal populations that express CB_1_Rs by showing that *Pvalb*^+^ and *Sst*^+^ interneurons that are unlikely to express CB_1_Rs (106) also undergo reorganization, with their increased numbers likely indicative of a circuit-level compensation to maintain inhibitory drive. This is plausible because both *Cck*^+^ and *Pvalb*^+^ interneurons target the perisomatic domain of pyramidal cells (107). Thus, and even though they are unlikely to confer equal network drive and flexibility, the increased number of *Pvalb*^+^ interneurons in hippocampal CA1 could be an attempt to stabilize inhibition/excitation balance (108).

Next, we show that THC exposure of healthy juvenile mice induces long-lasting changes at the level of their brain proteome, which endure into the adulthood of drug-exposed subjects. Here, we opted for a sampling paradigm, which specifically focused on long-term changes in protein expression and availability. Notably, and in comparison with proteomics data upon THC exposure in utero (11), we find that 12.5% and 28.1% of embryonic targets are also detected on P48 and P120, respectively. Thus, THC effects on pre- and postnatal neuronal development likely impinge upon some of the most fundamental processes that establish neuronal morphology and connectivity. Notwithstanding, energy demands of any developmental process underpinning a change in cell shape, size, position, and interactions are among the highest throughout a lifetime. This is particularly relevant to neurons whose energy demand to complete the membrane expansion and cytoskeletal reorganization required for axonal and dendrite growth is substantial (109, 110). The ability of ATP production of any cell upon extracellular stimuli depends on the ability of its mitochondria to dynamically adjust their number (initiating general growth, fission, or fusion; ref. 111), volume, and formation of cristae (110, 112, 113). Here, we find that THC increased the load of OXPHOS proteins together with the outer mitochondrial import protein TOM20, which implies, beyond an increased demand of ATP production, an increase in mitochondrial membrane surface. Therefore, increased mitochondrial size and/or number might be seen as compensatory mechanisms to fend off metabolic stress at a critical time of brain maturation.

Nevertheless, mitochondria are clearly more than just fuel cells of neurons; by organizing Ca^2+^ signaling through cation sequestration (97) and stimulus-dependent release, they directly regulate cytoskeletal dynamics (111, 114) and cell survival through the release of proapoptotic factors (115). If THC disrupts neuronal bioenergetics in developmental settings by either blocking the synthesis and translocation of mitochondrial proteins or occluding OXPHOS, as seen in adults acutely (40, 41), then one would expect that affected mitochondrial proteins be upregulated to compensate energy shortage. Indeed, we find — in accordance with recent studies on bioenergetic effects of THC in both neurons and glia (41, 74) — that essential building blocks of the complexes I–V mitochondrial complexes undergo significant upregulation, a change that persists until adulthood.

Our in vitro studies uncover a significant CB_1_R-independent component of THC action upon neuronal membrane integrity (stiffness and viscoelasticity as measures of fluidity) that defines survival. This is compatible with the more indiscriminate reorganization of the juvenile hippocampus in response to THC than previously thought. A CB_1_R-independent component of THC action that regulates mitochondrial bioenergetics (or at least the exclusion of cell surface CB_1_Rs from this process) can differentiate cell survival from merely curtailing neurite outgrowth, with the latter phenomenon being secondary in relative importance. Therefore, CB_1_R-driven cytoskeletal dynamics might succumb to energetic stress and/or imbalanced mitochondrial Ca^2+^ regulation.

A direct measure of mitochondrial stress is the disruption of the MMP, a crucial component of the mitochondrial proton driving force to produce ATP (and reactive oxygen species; ref. 116). Substantial and rapid collapse of the MMP, as seen in the presence of 7.5 μM THC, will inevitably lower intracellular pH and consequently eliminate the proton driving force. The ensuing ATP deprivation of neurons can ultimately trigger the sequelae of programmed death (117). This is why we have reasoned that any mechanism that can rescue intracellular pH, ion homeostasis, or lower energy expenditure (e.g., pyruvate) could counteract adverse THC effects. Indeed, both glutathione, an antioxidant that eliminates peroxy radicals (118), and TR019622 (olesoxime), a neuroprotective agent that acts at the level of VDAC, could produce near-maximal rescue of the MMP, neuronal survival, and neurite outgrowth. Beneficial TR019622 effects are plausible because VDACs bidirectionally control ion fluxes into and out of the mitochondrial matrix (97, 119), with a direct effect on the electrogenic gradient of the MMP (120). All 3 subunits of the VDAC were found upregulated in our proteomic analysis. VDACs act as lipid sensors (98) and can also be modulated by phytocannabinoids (99), interactions that can provoke cell death (98, 121). TR019622 is a small, lipophilic molecule with cholesterol-like structure (100, 122, 123), which allows the compound to interact with (122) and stabilize membrane integrity (101, 122). Thus, we find a THC-VDAC interaction plausible, which could directly be antagonized by TR019622. Another benefit of TR019622 application is its efficient inhibition of both caspase activation and cytochrome c release (101), key mediators of apoptotic cell death. Thus, our study reconciles detrimental THC effects, their molecular mechanism, and neuroprotective strategies that might also prove beneficial in vivo in the future.

Recently, the Marsicano group placed CB_1_Rs into the outer membrane of mitochondria and implicated its endocannabinoid and THC-driven activation in both neuronal (41) and glial respiration (74). Herein, a particularly strong effect on the mitochondrial complex I and sAC activation was suggested by using both pharmacological and genetic tools. KH7 is a sAC inhibitor, whereas HCO_3_^–^ is potent in activating sAC. As such, besides cAMP production, HCO_3_^–^ also stimulates ATP production and prevents mitochondrial swelling (94–96, 124). Here, we find that KH7 does not affect THC-induced changes in cellular bioenergetics while being detrimental for neuronal survival (data not shown). In contrast, HCO_3_^–^ significantly protected against THC toxicity. Nevertheless, in developing neurons probed here, HCO_3_^–^ effects seemed to be CB_1_R independent if one considers that AM251 could act intracellularly (85) and that HEK293 cells lacking CB_1_Rs (125) were also sensitive to THC-induced mitochondrial disruption. Based on our results, it is exciting to entice future studies addressing brain bioenergetics in translational settings and connecting energy availability and expenditure of the developing human brain to cognitive and academic performance in children exposed to THC.

THC not only shows complex pharmacological interactions, but also accumulates in neuronal membranes due to its lipophilic character. This propensity has direct relevance for cellular physiology by modulating membrane fluidity (45, 46). Since the biophysical properties of the cell membrane define, among others, the rate of exocytosis, mosaics of receptor multimers, and large signaling units and lipid rafts, one might hypothesize that THC-driven changes in membrane biophysics could lead to the collapse of ion channel–, receptor-, and transporter-dependent signaling events (126–128). The reduced membrane stiffness and altered viscoelastic properties determined by nanoindentation provide a direct measure of THC effects in intact neurons and support non–receptor-mediated THC effects that go beyond classical pharmacology. We have used an indentation depth of 1 μm to distinguish plasmalemmal changes without cytoskeletal confounders (92). Based on the lipophilicity of THC, we would quite certainly entertain the possibility that similar changes in membrane fluidity might also occur in intracellular organelles, most notably mitochondria, when THC concentrations are high. Thereby, THC enrichment of the mitochondrial membrane could in itself perturb the dynamics of VDAC-mediated anion fluxes (129). Thereby, we arrive to a complex cellular model in which CB_1_R-dependent cell surface and intracellular signaling events could drive OXPHOS and cellular respiration on the premise that THC incorporation in biological membranes and their altered fluidity can superimpose an added and CB_1_R-independent level of regulation. Given that these mechanisms invariably impinge on VDACs, they emphasize the relevance of TR019622-induced cytoprotection against THC toxicity.

In summary, we use a top-down approach encompassing neuronal systems-to-molecular biology to identify the molecular mechanisms by which THC impacts neuronal survival and circuit specification in the healthy juvenile brain. The THC concentrations used here seem relevant to recreational exposure in adolescents and preadolescents, especially considering the steadily increasing concentrations in cannabis preparations (2, 130). Accordingly, it is also highly unlikely that the THC concentrations used here could be inadvertently reached by approved therapeutic drugs (e.g., cannabidiol-based products). Notwithstanding, THC effects might be substantially different in a disease context when the brain is subject to neuronal disorganization, misrouted differentiation, or wiring due to a preexisting pathology. In case endocannabinoid signaling is affected by the pathomechanisms, THC use might be beneficial (e.g., endocannabinoid signaling is reorganized in Alzheimer’s disease, and low-dose THC treatment rescues dementia-like behavioral deficits in mouse models; refs. 131, 132). Ultimately, and considering the recent introduction of plant-derived and synthetic preparations with extraordinarily high THC contents (130), we caution against their use in either pediatric care or upon incidental exposure of healthy children. The finding that THC effects endure into the adulthood of affected juvenile rodents and impact cellular bioenergetics through a number of parallel pathomechanisms emphasizes the fundamental nature of developmental errors that a child’s brain might incur upon THC exposure.

## Methods

### Drugs.

pTHC (314.46 mg/mol, 95% purity) was provided in ethanol by GW Pharmaceuticals. sTHC was obtained from THC Pharm (diluted in methanol with 98.9% purity or ethanol with 99.4% purity) and from Lipomed (in ethanol with > 97% purity). Drug preparations and treatment regimens are referred to in the Supplemental Methods (SM).

### Study design and approval.

Male *Cck*^BAC/DsRed^ (65) (gift of F. Erdélyi, G. Szabó, and Z. Máté, Institute of Experimental Medicine, Budapest, Hungary), *Cck*^BAC/DsRed^*Gad1*^gfp/+^ mice (66) (*Gad1*^gfp/+^ were donated by Y. Yanagawa, Gunma Medical School, Japan), or C57BL/6J mice were used as described in the SM. Experiments on live animals conformed to the 2010/63/EU European Communities Council Directive and were approved by the Austrian Ministry of Science and Research (ethical permit numbers 66.009/0145-WF/II/3b/2014 and 66.009/0277-WF/V/3b/2017). Particular effort was directed toward minimizing the number of animals used and their suffering during experiments.

### Radioligand binding.

[^3^H]CP55,940 (American Radiolabeled Chemicals) and [^3^H]SR141716A (PerkinElmer) radioligand binding to C57BL/6J mouse cortices at E18, E5, P2, P16, and adulthood were performed as described (133) (see also SM).

### Quantitative histochemistry and light-sheet microscopy.

For IHC, sections were processed according to standard protocols (60, 134) with immunoreagents listed in Supplemental Table 1. Intact tissue imaging by light-sheet microscopy was undertaken as described in ref. 135 (see SM for details).

### Behavior.

Spontaneous animal behaviors a day before sample collection for iTRAQ proteomics to confirm no acute THC effects as described in ref. 136.

### iTRAQ proteomics and Western blotting.

Batches of hippocampi (right side) were processed on either P48 (midadolescence; *n* = 5/group) or P120 (adulthood; *n* = 5 per group) in 8-plex iTRAQ runs (see SM) according to published protocols (11, 137). The left hippocampus of each mouse was used for Western blotting, with primary antibodies listed in Supplemental Table 1.

### Neuronal cultures and IncuCyte imaging.

Neurons were isolated from E14.5 C57BL/6J mouse cortices (60) and imaged in an IncuCyte live-cell imaging device (Essen Bioscience) as described in the SM.

### Mito-ID.

The Mito-ID Membrane Potential Detection Kit (ENZ-51018, Enzo Life Sciences) was used to determine the MMP of cortical neurons as described in the SM.

### Nanoindentation.

Neurons at a density of 500,000 cells/well were tested 1 hour after the start of drug challenges using a Chiaro nanoindentation instrument (Optics11; refs. 138–140) (see SM for details).

### Data availability.

Proteomic data are being deposited in PRIDE (accession no. PXD010802) and will be made public.

### Statistics.

Data are expressed as mean ± SD or SEM as stated. Dose-response relationships and saturation in radioligand binding experiments were determined by nonlinear regression analysis using Excel (Microsoft) and GraphPad Prism (GraphPad). Bartlett’s test was used by default to assess homogeneity of data. Neuronal distribution of c-Fos^+^ cells was evaluated by Student’s 2-tailed *t* test (GraphPad), and density on P16 was evaluated by 1-way ANOVA with Bonferroni’s post hoc correction (GraphPad). For iTRAQ proteomics, our experimental design asked if changes exist for vehicle versus 1 mg/kg pTHC versus 5 mg/kg pTHC. Statistical analyses of filtered protein targets across treatment groups were performed in SPSS (21.0) using 1-way ANOVA comparisons, including Levene’s test for homogeneity followed by Bonferroni’s correction. Data from Western blotting and MMP were evaluated using ANOVA comparisons followed by Bonferroni’s correction (GraphPad). IncuCyte data were processed manually using the last pretreatment point as baseline, with endpoint data referenced as percentage change. Statistical analysis was conducted in GraphPad Prism using 2-way ANOVA comparisons followed by Bonferroni’s correction (GraphPad). *P* < 0.05 was considered statistically significant. Accordingly, and to ease the readability of this report, only *P* value*s* from pairwise comparisons where the main effect ANOVA value was significant were reported in Results.

## Author contributions

TH, EK, VDM, RG, and GWG conceived the project; JB, ZH, DC, JP, RAR, ES, GL, SS, CHB, RG, and EK performed experiments and analyzed data; and JB, EK, SK, and TH wrote the manuscript with input from all coauthors.

## Supplementary Material

supplemental data

## Figures and Tables

**Table 1:**
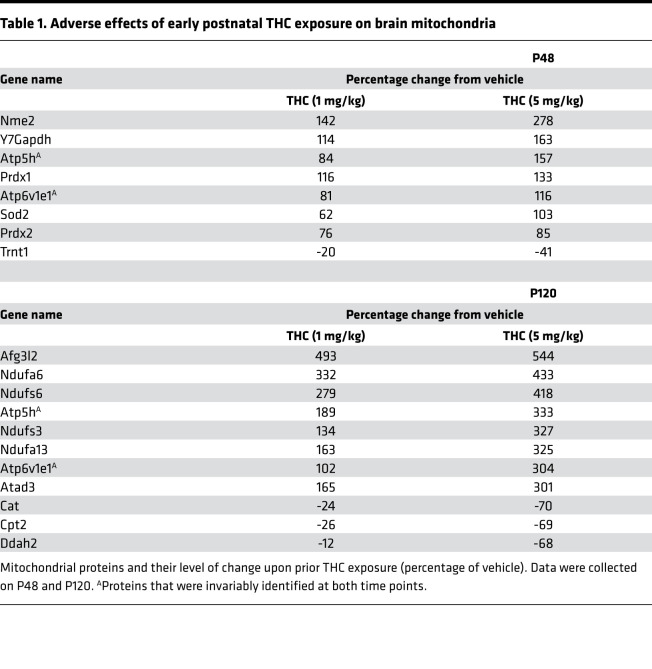
Adverse effects of early postnatal THC exposure on brain mitochondria

**Table 2:**
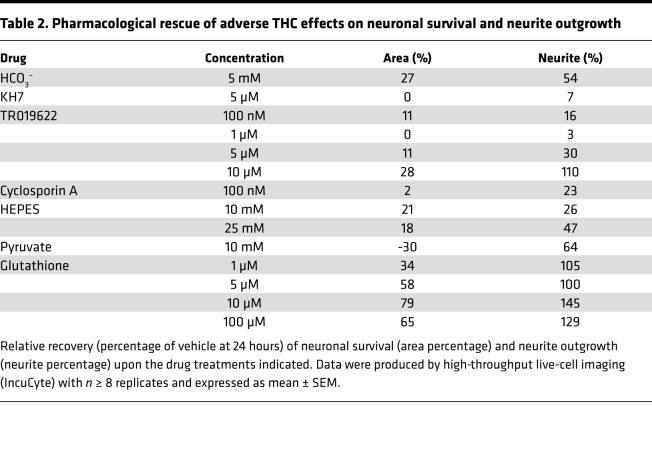
Pharmacological rescue of adverse THC effects on neuronal survival and neurite outgrowth

## Version 1 11/03/2020

In-Press Preview

## Version 2 12/03/2020

Electronic publication
