# Supplementary material for: Adverse effects of Δ9-tetrahydrocannabinol on neuronal bioenergetics during postnatal development
Source: JCI Insight. 2020 Dec 3;5(23):e135418. doi: 10.1172/jci.insight.135418 (PMC7714410; doi:10.1172/jci.insight.135418)
Supplement: supplemental data [file jciinsight-5-135418-s030.pdf]

Revision to manuscript **#135418-INS-RG-TR-3**:

Supplementary Information to the manuscript:

Adverse effects of Δ^9-tetrahydrocannabinol on neuronal bioenergetics during postnatal development

Johannes Beiersdorf, Zsofia Hevesi, Daniela Calvigioni, Jakob Pyszkowski, Roman A. Romanov, Edit Szodorai, Gert Lubec, Sally Shirran, Catherine H. Botting, Siegfried Kasper, Geoffrey W. Guy, Roy Gray, Vincenzo Di Marzo, Tibor Harkany and Erik Keimpema

This file contains:

- Supplementary methods,
- Supplementary figures and legends,
- Statistical spreadsheets for 1-S9,
- Supplementary table with legend,
- Supplementary references.

Supplementary methods

Drugs

Plant-derived, highly-purified THC (pTHC; 314.46 mg/mol, 95% purity) was provided in ethanol by GW Pharmaceuticals. Synthetic THC (sTHC) was obtained from THC Pharm (diluted in methanol with 98.9% purity or ethanol with 99.4% purity) and from Lipomed (in ethanol with > 97% purity). For drug preparations, ethanol/methanol was evaporated in a N_2 gas flow to limit oxidization. sTHC was subsequently diluted and aliquoted in dimethyl sulfoxide (DMSO; Sigma) and stored at -80°C. For *in vitro* applications, the stock solutions were mixed in an ultrasonic bath before being further diluted in corresponding concentrations of cell culture media for experimental use. pTHC was either diluted in DMSO for *in vitro* and pharmacology applications following the same strategy as for sTHC or further diluted and aliquoted in ethanol for the preparation of *in vivo* applications. For *in vivo* application, the ethanol of pTHC was evaporated in a N_2 gas flow and the substance was diluted freshly in a mixture of physiological saline (sterile, Mini-Plasco®, Braun) and 3 v/v% Tween 80 (Sigma) as emulsifier. After ultrasonication to reach complete dissolution, a volume of 100 µl was injected subcutaneously (s.c.) immediately on each day from P5-16 or P5-35 (*see below*). pTHC at 1 or 5 mg/kg bodyweight was used (1), which when employing the FDA allometric scaling guidance (https://www.fda.gov/media/72309/download) corresponds to 24.3 mg/60 kg bodyweight. This dose is in the range of exposure for regular cannabis smokers when considering 5-15% THC content in regular herbal cannabis preparations (2), which results in 50-150 mg THC/g cannabis. Even higher THC intake seems relevant for cannabis resin and high potency extracts.

The control group received an equivalent volume of the vehicle solution daily. Drug doses and injection volumes were adjusted to the bodyweight of the animals, which was monitored every other day (*data not shown*). Notably, juvenile offspring was injected directly thus this model was neither intended to test maternal-to-offspring drug transfer nor bioavailability upon inhalation or digestion.

For ligand binding at CB1Rs, pTHC and sTHC in DMSO were ultrasonicated and diluted in 50 mM Tris-HCl buffer (pH 7.4) also containing 0.1% bovine serum albumin (BSA). For *in vitro* studies, stock solutions of plant-extracted or synthetic THC in DMSO were ultrasonicated and diluted directly in cell culture media and used freshly. An identical procedure (including initial solvent evaporation in N_2 flow and preparation of DMSO stocks) was used for AM251 (1 µM; Tocris), O-2050 (1 µM; Tocris), TRO19622 (10 µM; Tocris), KH7 (5 µM; Tocris) and cyclosporin A (100 nM; Tocris; *final concentrations* were given). Sodium bicarbonate ($NaHCO_3$, Sigma) and 4-(2-hydroxyethyl)-1-piperazineethanesulfonic acid (HEPES; GIBCO®, Life Technologies) stocks were prepared in H_2O and diluted in cell culture medium.

Radioligand binding

C57Bl6/J mouse cortices at embryonic day (E)18,5, P2, P16 and adulthood were dissected and homogenized with a glass/teflon Potter-type homogenizer in ice cold 50 mM Tris-HCl buffer (pH 7.4) also containing 3 mM ethylenediaminetetraacetic acid (EDTA). Total membranes were fractionated at 35,000*g* for 10 min followed by a washing step, repeated centrifugation and pellet re-suspension in 50 mM Tris-HCl buffer. Intracellular constituents were removed from re-suspended membrane preparations by incubation at 23 °C for 2h before suspensions were centrifuged, re-suspended, aliquoted and stored at -80°C. BSA at a concentration of 0.1% was used throughout the experiments in order to retain both [^{3}H]CP55,940 (American Radiolabeled Chemicals) and [^{3}H]SR141716A (PerkinElmer) in solution and protect them from being precipitated on glass surfaces (3). $MgCl_2$ (3 mM) was used to increase binding specificity. Binding experiments followed a displacement design with set radioligand concentrations challenged by increasing concentrations of unlabeled ligands. Non-specific binding was determined for immoderately high ligand concentrations. The composite was incubated at 23 °C for 3h prior to being rinsed over glass fiber filters (Watman GF-C, presoaked in 0.3% polyethylene) in a vacuum-driven Hoefer filtration box. Filters were washed (3x) with 50 mM Tris-HCl buffer before being exposed to a scintillation cocktail (Rotiszint 11; Roth) for 20 mins and measured in a Packard TRI-CARB 2100TR scintillation counter. Experiments were performed in triplicate. Protein concentrations of the samples were determined by the Bradford method (Bio-Rad (4)).

Animals, tissue preparation and quantitative histochemistry

Male *Cck*$^{\mathrm{BAC/DsRed}}$ (provided by Dr. Ferenc Erdélyi, Dr. Gábor Szabó and Dr. Zoltán Máté, Institute of Experimental Medicine, Budapest, Hungary (5)), *Cck*$^{\mathrm{BAC/DsRed}}$::*Gad1*$^{\mathrm{gfp/+}}$ mice (given by Y. Yanagawa, Gunma Medical School, Japan (6)) or C57Bl6/J mice were used as indicated. pTHC (1 mg/kg or 5 mg/kg) or vehicle were administered daily from P5 to P16 with relation to human dose equivalents as above. Animals were perfusion fixed with 4% paraformaldehyde (PFA, 50 ml) diluted in 0.1M phosphate buffer (PB, ph 7.4) at a flow rate of 2-4 ml/min, which was preceded by a brief infusion of ice-cold physiological saline. Subsequently, brains were dissected out, postfixed in 4% PFA overnight, cryoprotected in 30% sucrose in PB for >48h and sectioned coronally at 50 μm thickness on a Leica CM1850UV cryostat microtome and processed free-floating throughout (7). The number of animals was reported in the figure legend for each experiments separately.

For immunohistochemistry, sections were rinsed in 0.1M PB before being exposed to a cocktail of 5% normal donkey serum (NDS; Jackson ImmunoResearch), 1% BSA (Sigma), and 0.3% Triton X-100 (Sigma) in PB at 22-24 ^{o}C for 1h to quench non-specific immunoreactivity. Tissues were then exposed to primary antibodies (Table 1) diluted in PB supplemented with 0.1% NDS and 0.3% Triton X-100 at 4 °C for 48-72h. After extensive rinsing, the specimen was exposed to secondary whole IgGs raised in donkey and tagged with DyLight Fluor 488, 560 or 633 fluorochromes (1:300; Jackson

ImmunoResearch; 22-24 °C for 2h). Hoechst 33,342 (Sigma) was routinely used as nuclear counterstain. After repeated rinses in PB and a brief de-salting step in distilled water, sections were mounted on fluorescence-free glass slides, air-dried and coverslipped with Entellan (in toluene, Merck). Single-channel images were captured on a Nikon Microphot FXA Epi-FL3 epifluorescence microscope equipped with a PCO-Pixelfy monochrome camera. Multicolor images were acquired on a Zeiss 880LSM confocal laser scanning microscope. Cell counts as well as surface and distance definitions were performed in ImageJ 2006.02.01 including Fiji applications. In each animal, $n = 3$ sections spanning the dorsal hippocampus with an inter-section interval not exceeding 200 μm were analyzed, and included cell counts in both hemispheres. The same protocol was used for c-Fos^{+} cell counts in the amygdala, aimed to determine neuronal activation on P9 (Figure S1D,D_1). To determine the spread of pyramidal cells (as a function of cell survival after THC treatment), $n = 9$ measurements per hemisphere were conducted by measuring the vertical distance between the most dorsal and ventral cells in CCK$^{BAC/DsRed}$ reporter mice.

Intact tissue imaging by light-sheet microscopy

Cck$^{BAC/DsRed}$::*Gad1*$^{gfp/+}$ brains (P16) were used for optical tissue clearing by a modified CUBIC protocol (8) and subsequent light-sheet microscopy. After post-fixation in 4% PFA, brains were transferred to CUBIC 1 solution (25% urea, 25% *N,N,N',N'*-tetrakis-(2-hydroxypropyl)ethylenediamine and 15% Triton X-100; all from Sigma) for 6 days at 37 °C while shaking. CUBIC 1 solution was prepared freshly and replaced after 3 days. After extensive washing (3x 30 min in 0.1M PB), the samples were immersed in CUBIC 2 solution (50% sucrose, 25% urea, 10% *2,2',2''*-nitrilotriethanol and 0.1% Triton X-100; all from Sigma) for another 6 days. Images were acquired on a Lightsheet Z.1 microscope (Zeiss) with the samples immersed in CUBIC 2 solution to keep a refractory index of 1.45 while imaging. Each optical plane was illuminated from a single side with x5 (EC Plan Neofluar 5x/0.16) objective and laser excitation at 488 and 561 nm. A protocol of x0.7 zoom with individual z-stack intervals of 2.5µm was set. Laser power was limited to 20% with exposure times of 200 ms. Three-dimensional images were acquired through tile scanning with their brightness and contrast adjusted manually to id visual clarity within the Arivis Vision 4D for Zeiss (v.2.12) software package.

Behavior

We tested spontaneous animal behaviors a day prior to sample collection for iTRAQ proteomics to confirm no acute THC effects. We chose the elevated plus-maze test that is frequently used to assess anxiety-like phenotypes brought about by psychoactive drugs (9). Each session lasted for 4 min under dimmed lighting conditions. Video records were analyzed off-line (Panlab, Smart Video Tracking Software 3.0.03).

iTRAQ proteomics

Male C57Bl6/J mice were treated with pTHC (1 mg/kg or 5 mg/kg) or vehicle from P5 to P35. At P25, all animals were weaned, but remained group-housed for the duration of the study. Food and water were available *ad libitum*. The first batch of animals was sacrificed by decapitation on P48 ('mid-adolescence'; *n* = 5/group), whereas the second batch was processed on P120 ('adulthood'; *n* = 5 per group). Both hippocampi were dissected, flash-frozen in liquid N_2 separately and stored at -80 °C. iTRAQ was performed on the *right* hippocampus of each subject according to published protocols (1, 10) in 8-plex runs (iTRAQ AB Sciex Pte.). Samples were ordered to keep maximal intra- and inter-group distances, and to minimize sampling bias. A control sample (vehicle-treated mouse, designated as 'vehicle 1') was repeatedly loaded on all iTRAQ chips (per time-point) to aid cross-comparisons and sample normalization. The arrangement of samples (including specificity tags) across the multiple 8-plex layouts was kept consistent for both time-points. The data generated from the individual fractions of each sample were combined and analyzed using Sciex Protein Pilot v4.0. Data were searched against the SwissProt database (downloaded Jun2015) restricted to mouse only proteins. Target peptides were first filtered for a confidence interval above 99% and thereupon selected using the ProteinPilot software. This procedure was performed separately for each of the iTRAQ layouts. Subsequently, protein levels above the threshold were filtered for common proteins which occurred in each of the chips. At P48, 2,076 proteins were consistently identified. For P120, 3,542 commonly-occurring proteins were found. All logscale data-points were normalized to 'vehicle 1'. The description and classification of proteins is based on the gene ontology (GO) database www.uniprot.org.

Western blotting

The *left* hippocampus of each mouse was lysed and homogenized in HEM^+-buffer (25mM HEPES, 1mM EDTA, 6mM $MgCl_2$, 1mM DTT, 1x protease inhibitor) before preparing membrane and cytosolic fractions. Initial centrifugation at 500 *g* at 4 °C for 5 min was performed to remove nuclei and cellular debris. Cytosolic fractions were obtained after additional centrifugation at 14,400 *g* at 4°C for 30 min. Membrane fractions were isolated after an additional centrifugation step (14,400 *g* at 4°C for 30 min) by re-suspension of the resultant pellet. Protein concentrations were determined by the Bradford assay (Bio-Rad (4)). For protein analysis, 10 µg of each sample was incubated with Carbocyanine 5 (Cy5; GE-Healthcare) for 30 min prior to the addition of loading buffer (GE-Healthcare). Denaturation at 95° for 5 min was optionally performed. Protein probing was performed under denaturing conditions on 13.5% SDS gels before transferring the proteins onto PVDF membranes (both from GE-Healthcare). Samples were incubated with primary antibodies (Table 1) at 4°C overnight after a blocking step (3% BSA in 0.05M Tris-buffered saline, pH7.4). Secondary antibodies were conjugated to Cy3 (GE-Healthcare, 1:5,000; 1h incubation). An automated Amersham Western Blot system (GE-Healthcare) was used to visualize Cy3 and the Cy5 signals.

Neuronal cultures and IncuCyte imaging

Neurons were isolated from E14.5 C57Bl6/J mouse cortices (11) and grown in Neurobasal A medium (GIBCO, Life Technologies) supplemented with L-glutamine (2 mM), B27 supplement (2%) and penicillin-streptomycin (1%). Cells were plated in 96-well plates (25,000 cells/well; 0030730119, Eppendorf) pre-treated with poly-D-lysine in 0.1M borate buffer overnight. Drug challenges were initiated on day 2 *in vitro* (DIV). Antagonists and rescue protocols were applied 30 min before THC application. An IncuCyte live-cell imaging device (Essen Bioscience), itself placed in an incubator with stable 5% CO_2 intake and temperature control at 37 °C, as used for live cell (including neurite) imaging. Phase contrast images were taken at hourly intervals (or every other hour). 'The area of cell viability', that is the surface area occupied by cell bodies (mm^2/mm^2) was taken as a measure of cell viability. The growth rate of neurites was obtained by using the 'neurite length' mask (mm/mm^2). Optionally, neurite branching was also defined as branch points per surface area (n/mm^2; *data not shown*). All measures were optimized in preliminary experiments, including drug doses and treatment paradigms. *In vitro* imaging of antibody-labeled primary neurons was conducted on an LSM 880 confocal laser scanning microscope (ZEISS), while super-resolution microscopy of TOM20-stained mitochondria was performed on an ELYRA PS.1 system (Zeiss) as previously described (12).

Mito-ID

To determine the MMP of Hek293 cells (American Type Culture Collection, ATCC) and cortical neurons, these (E14.5) were plated at a density of 200,000 cells/well in poly-D-lysine-coated 4 compartment glass bottom dishes (#627870; Greiner Bio-One) with treatments commencing on 2DIV. MMP was measured using the Mito-ID® Membrane Potential Detection Kit (ENZ-51018; Enzo Life Sciences). All substances and buffers were prepared according to manufacturer's protocol. The length of pharmacological treatments as well as staining to visualize low and high MMP followed the manufacturer's recommendations. THC was applied 30 min before washing (2x) the cells with Mito-ID® Assay Buffer. Then, Mito-ID® MP Detection Reagent was applied for 15 min and subsequent measurements were performed in the MMP Assay Buffer in a humidified atmosphere (5% CO_2) at 37°C. Energized mitochondria with high MMP were detected by using a rhodamine filter (excitation at 540 nm, emission at 570 nm), whereas depolarized mitochondria with low MMP were detected with a fluorescein filter (excitation at 485 nm, emission at 530 nm). Images (n = 10/condition/experiment) were taken at random positions in looped sequences from control-to-treatment conditions. The ratio of high MMP over low MMP signal was determined. Signal intensity was measured for each channel separately using ImageJ.

Nanoindentation

Neurons were isolated from E14.5 C57Bl6/J mouse cortices (11) and maintained as above at a density of 500,000 cells/well in poly-D-lysine pre-coated 6-well plates (Falcon). On DIV4, membrane properties

(stiffness and viscoelasticity as measures of fluidity) were determined 1h after the start of drug challenges using a Chiaro nanoindentation instrument (Optics 11 (13-15)). For all measures, a spherical glass indentation probe with a spring constant of 0.2 N/m and a diameter of 37 µm was used to generate force onto the cellular surface with a controlled piezo displacement rate of 3 µm/s. While the cellular surface was stressed by the indentation glass tip, the resulting force and surface displacement of the cell were recorded. Simultaneous live imaging with an EVOS® cell imaging inverted microscope (Thermo Fisher) with integrated screen allowed to confirm the accurate positioning of the glass tip (*data not shown*). The Chiaro Nanoindenter as well as the microscope and samples were positioned on a vibration free table to maximize noise reduction. The effective Young's Modulus was modelled up to an indentation depth of 1 µm, within the linear elastic regime and the Hertz model was applied for the initial part of the loading curve, as suggested for testing biological viscoelastic materials (16, 17). The effective Young's Modulus Eeff is denoted as $E_{eff} = \frac{3}{4\,P\,R^{1/2}\,h^{3/2}}$, with R: indenter tip radius, h: indentation depth and P: indenter load. Cellular stress-response was measured by applying a constant indentation depth for 10 seconds and recorded was the opposing cellular force.

Supplementary figures & legends

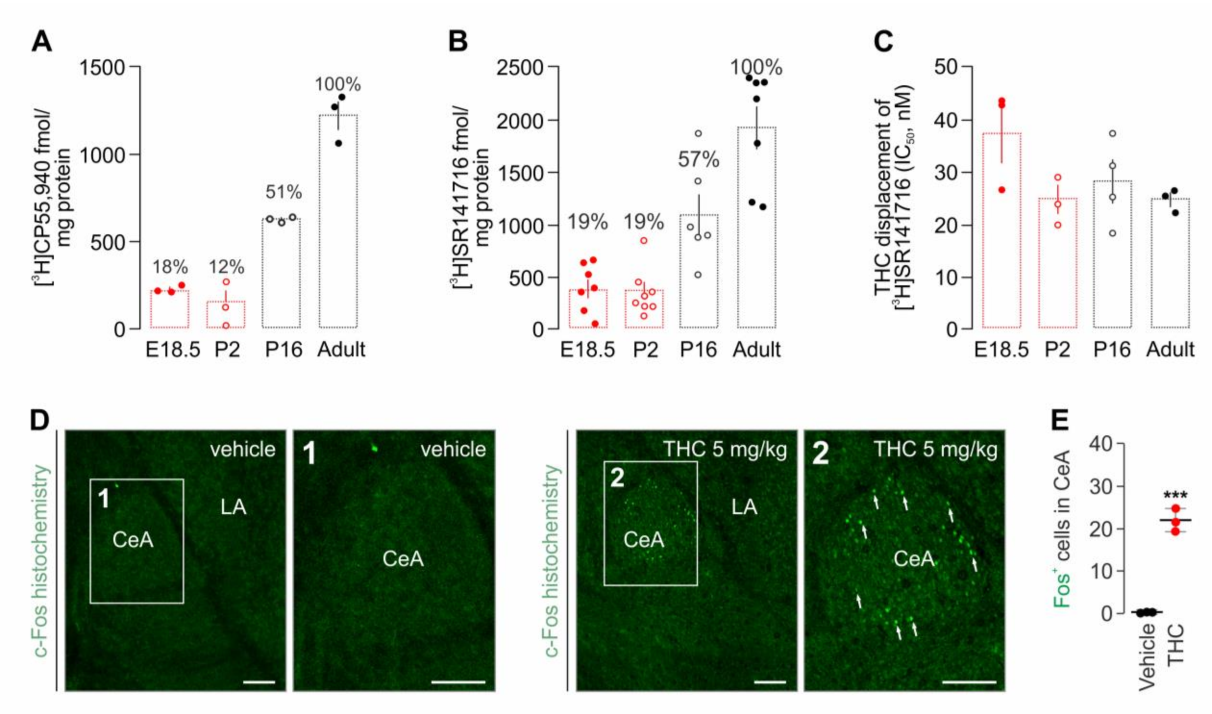

Figure S1. *CB1R radioligand binding during brain development and neuronal c-Fos activation upon acute pTHC exposure*. Saturation binding for both [^{3}H]CP55,940 (**A**) and [^{3}H]SR141716A (**B**) at defined developmental stages shows a gradual increase in CB1R binding sites (B_{max}) in the cerebral cortex (n = 3/developmental stage for [^{3}H]CP55,940 and n = 6-8/age group for [^{3}H]SR141716A). (**C**) Displacement of [^{3}H]CP55,940 by pTHC had unchanged IC_{50} throughout cortical development (n = 3-4/age group). (**D**) c-Fos histochemistry in juvenile mice (P9) exposed to vehicle (*left*) or 5 mg/kg THC (*right*) for 2h. Rectangles denote the central amygdaloid nucleus (CeA) shown in enumerated and high-magnification insets. (**D$_1$**) Quantitative data on c-Fos$^+$ cell numbers in the CeA upon vehicle or THC administration acutely (n = 3 mice/treatment). *$p < 0.05$, two-tailed Student's t-test. *Scale bars* = 100 µm. *Abbreviations*: E, embryonic day; LA, lateral amygdaloid nucleus; P, postnatal day.

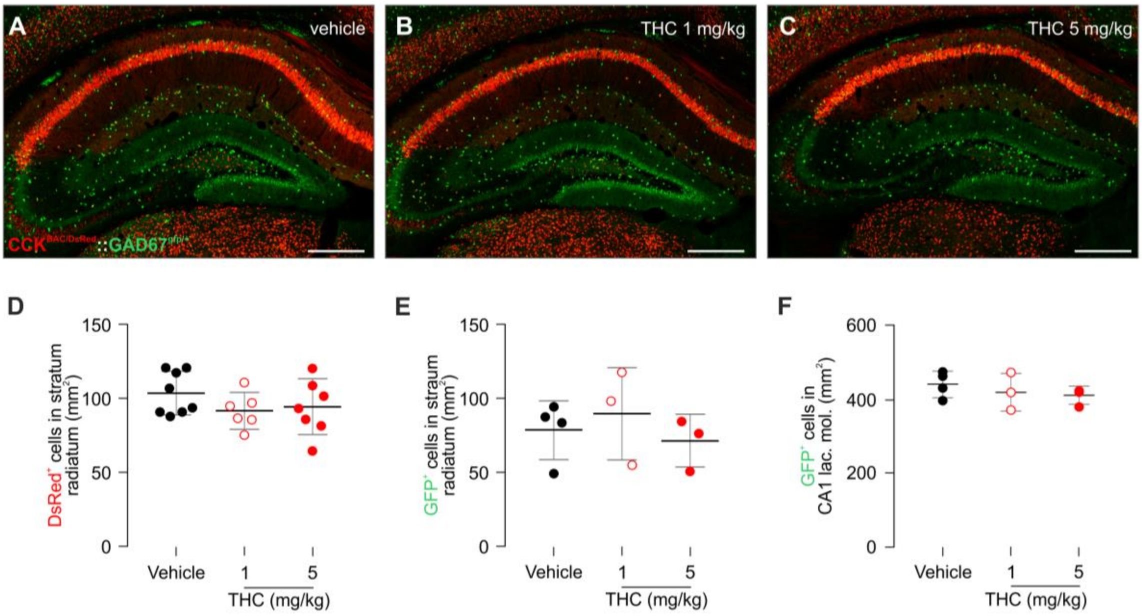

Figure S2. *Hippocampal architecture in Cck$^{BAC/DsRed}$::Gad1$^{gfp/+}$ mice on postnatal day (P)16 after repeated pTHC exposure*. (**A-C**) Representative multi-tiled images of the dorsal hippocampus captured by confocal laser-scanning microscopy after the treatments as indicated in overlay. Scale bars = 500 µm. (**D-F**) Quantitative analysis of DsRed$^+$ or GFP$^+$ neuronal densities in the CA1 subregions indicated on the *y*-axis, expressed as neurons/mm^2. Data were expressed as means ± s.d. (n = 3-8/genotype/treatment with n =3-4/treatment for *Cck*$^{BAC/DsRed}$::*Gad1*$^{gfp/+}$ mice) and analyzed by one-way ANOVA followed by Bonferroni's *post-hoc* correction for statistical significance (in all cases $p > 0.2$).

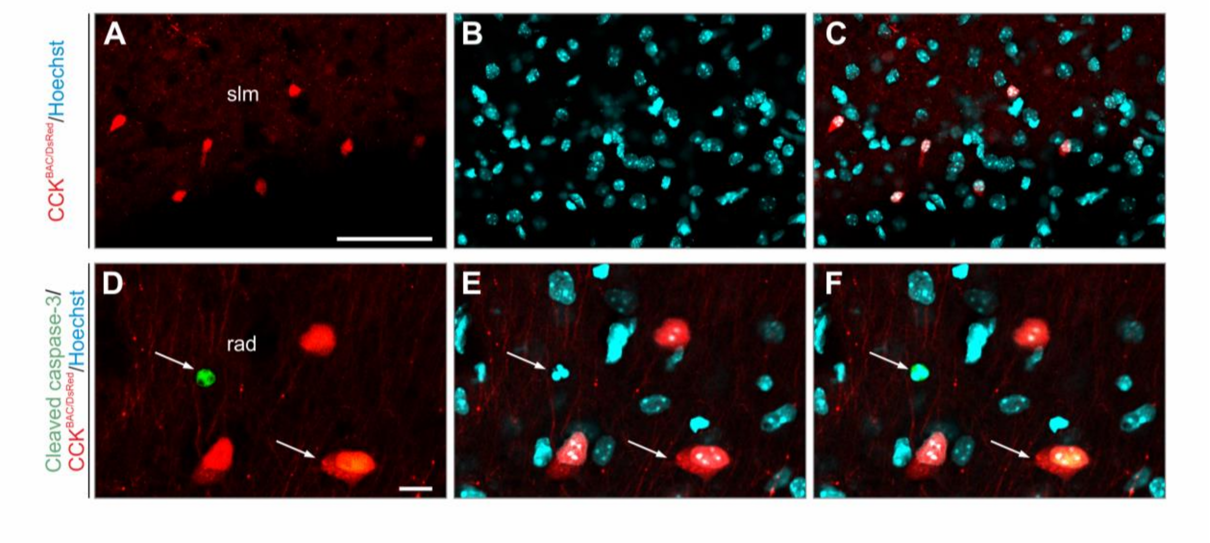

Figure S3. *DsRed⁺ neurons in non-pyramidal hippocampal layers do not show overt cleaved caspase 3 immunoreactivity upon THC exposure*. (**A-C**) Co-localization of DsRed and Hoechst 33,324 nuclear signal shows that even small-sized DsRed$^+$ puncta at the ventral-most border of the stratum lacunosum moleculare (slm) are *bona fide* neurons. (**D-F**) Cleaved caspase-3 immunoreactivity was scarce, randomly co-localized with DsRed and showed no difference between vehicle-treated and THC-exposed animals (irrespective of the THC dose) on P16. Arrows indicate cleaved caspase-3$^+$ cells in the stratum radiatum (rad) with/without DsRed co-localization whose fragmented nuclear structure (by Hoechst 33,324) suggests late-stage apoptosis. *Scale bars* = 100 µm (A), 10 µm (B).

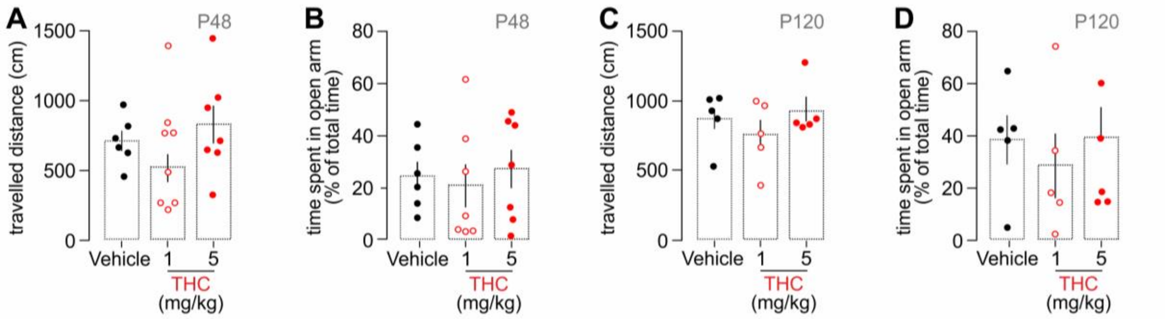

Figure S4. *THC exposure during preadolescence with a wash-out period of >14 days does not exert long-lasting effects on anxiety-like behaviors.* Mice had received vehicle or pTHC (at doses of 1 or 5 mg/kg) during the period of P5-P35 and tested either on P48 (**A,B**) or P120 (**C,D**). Anxiogenic phenotypes in THC-exposed mice, represented by reduced locomotion (**A,C**) and a reduced number of entries into the open arms of an elevated plus-maze apparatus (**B,D**), were not observed at either time point. Data were expressed as means ± s.e.m.; *n* = 5-7 mice/group.

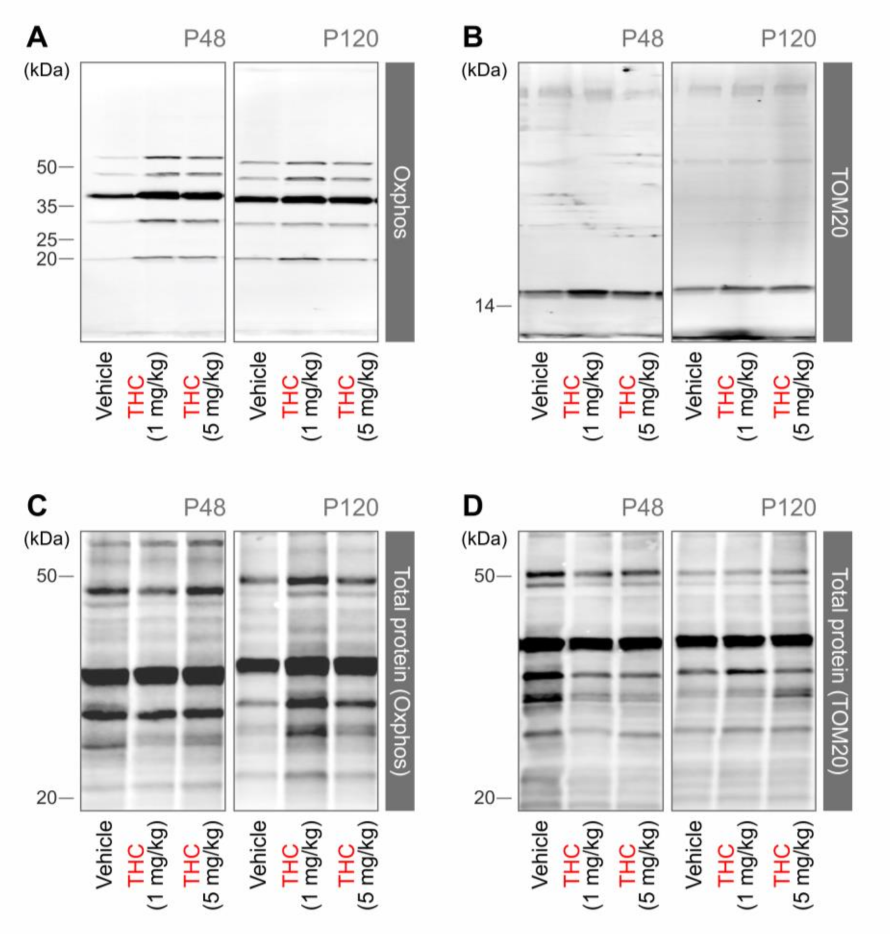

Figure S5. *Total protein load visualized with Cy5 and used to normalize and quantitate target proteins by Western blotting.* Representative Cy5 labeling results for total protein (**C,D**) load used to normalize key members of the oxidative phosphorylation machinery (OXPHOS; **A**) and the mitochondrial import receptor subunit TOM20 homolog (TOM20; **B**) expression on postnatal day (P)48 or 120 after 1 or 5 mg/kg pTHC treatment of juvenile mice (P5-P35). Quantitative data for OXPHOS and TOM20 are shown in Fig. 2C and 2F, respectively.

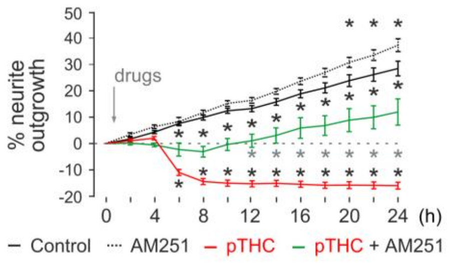

Figure S6. *THC-induced reduction of neurite outgrowth is CB1R dependent.* Time-lapse imaging of neurite growth upon exposure to 10 µM plant-derived THC (pTHC) was performed by using an IncuCyte Zoom device. The overlay mask used to trace neurite coverage of the sampled surface was manually adjusted for maximal signal separation. A loop time of 2h was used. THC induced rapid and significant neurite retraction and led to growth arrest by 24h. This effect was significantly rescued by AM251 (1 µM), a CB1R antagonist, which by itself induced neurite outgrowth. Black asterisks label statistically significant differences *vs*. control, while gray asterisks point to significant differences between THC *vs*. THC + AM251 groups. Data from $n \geq 8$ replicates were expressed as means ± s.e.m. and statistically evaluated by two-way ANOVA followed by Bonferroni's *post-hoc* correction; $^*p < 0.05$.

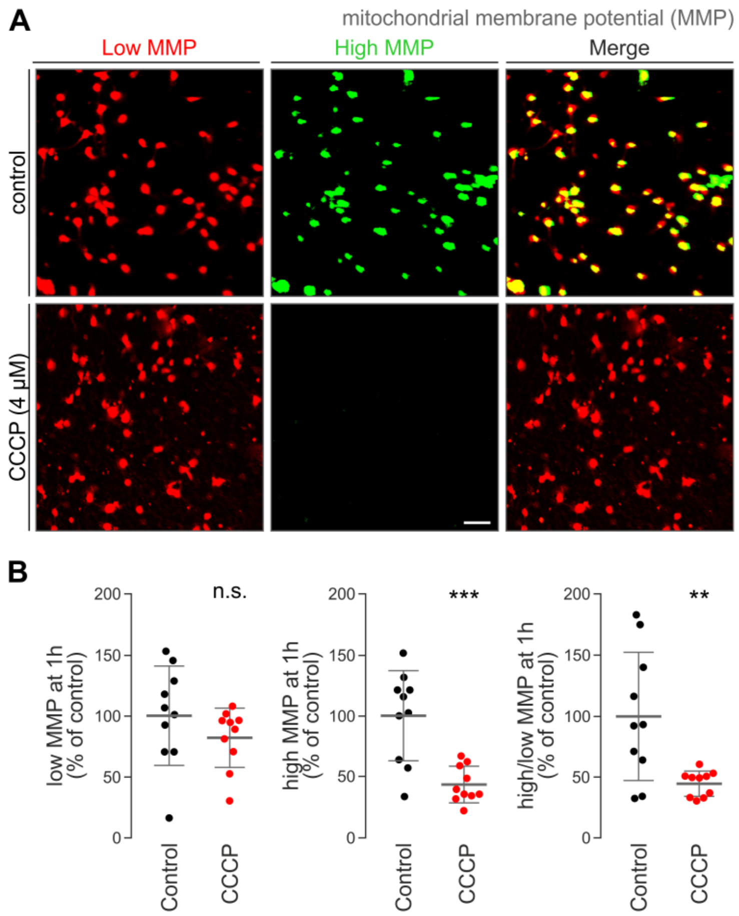

Figure S7. *Quality control of mitochondrial membrane potential (MMP) imaging in cortical neurons in vitro.* (**A**) Loss of high mitochondrial membrane potential upon application of carbonyl cyanide m-chlorophenyl hydrazine (CCCP; 4 µM), a protonophore (H^+ ionophore) and uncoupler of oxidative phosphorylation in mitochondria. Note the unchanged low MMP. *Scale bar* = 10 µm. (**B**) Quantitative ratios of aggregated fluorophore (high MMP) over monomeric fluorophore (low MMP) as measured by Mito-ID staining of live cells. CCCP, as positive control, indeed uncoupled mitochondria and reduced high MMP. Data points denote independent observations with means ± s.d. are also shown. Data were statistically evaluated by one-way ANOVA followed by Bonferroni's *post-hoc* correction; ns: non-significant, $**p < 0.01$, $***p < 0.001$.

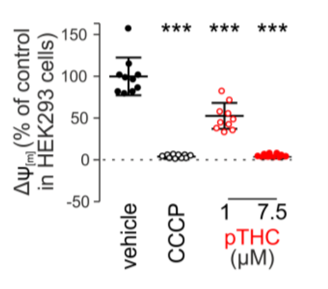

Figure S8. *Plant-derived THC disrupts the mitochondrial membrane potential in HEK293 cells lacking CB1R expression*. THC at a concentration of 7.5 μM completely abolished the mitochondrial membrane potential (MMP/Δψ) in HEK293 cells (ATCC), an effect that was similar in magnitude to that brought about by carbonyl cyanide m-chlorophenyl hydrazine (CCCP; 4 μM), used as positive control. In turn, 1 μM THC led to a significant yet partial reduction of the MMP. Data were expressed as means ± s.d. with the results of n = 10 replicates also shown. Data were statistically evaluated using one-way ANOVA (*vs*. control); ***p < 0.001.

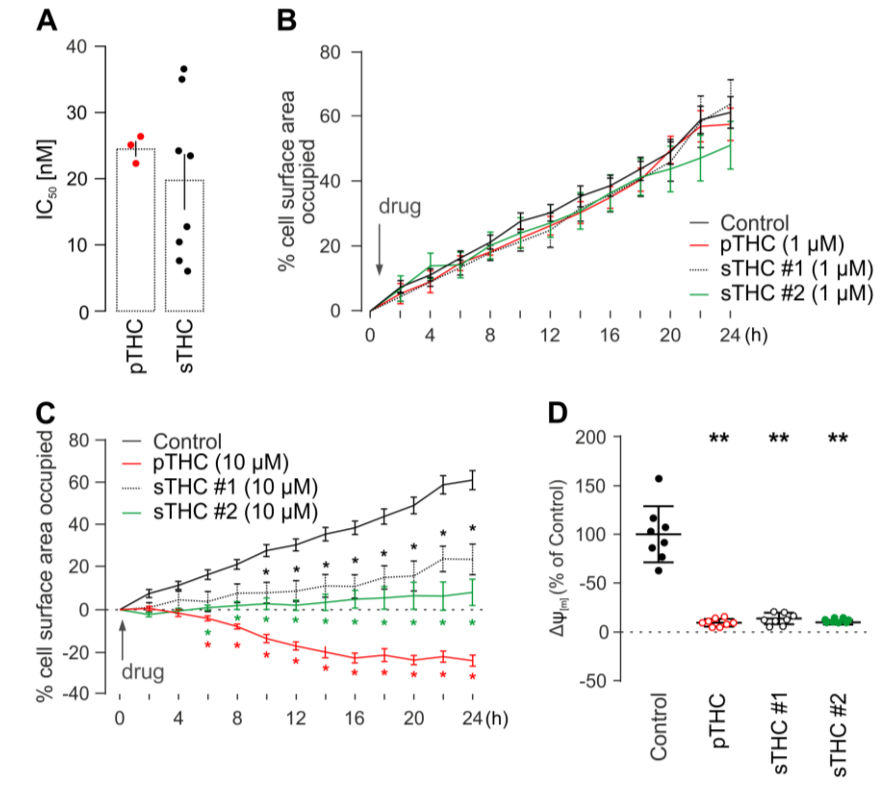

Figure S9. *Comparison of plant-derived and synthetic THC batches for in vitro use***.** (**A**) Displacement of [^{3}H]CP55,940 by plant-derived (pTHC) *vs*. synthetic THC (sTHC) did not reveal significant differences in IC_{50} in adult mouse cortical homogenates ($n \geq 3$/age group). (**B**) Dynamic monitoring of cell growth by an IncuCyte Zoom imaging system shoed no effect of either pTHC or sTHC (2 batches, independent vendors) at 1 μM concentration. (**C**) In contrast, both pTHC and sTHC (both batches) induced significant growth retardation at 10 μM concentration *in vitro*. Note that while sTHC arrested neuronal growth, pTHC led to neuronal death as indicated by the negative growth curve. Data in (**B,C**) were expressed as means ± s.e.m. Colored asterisks label statistically significant differences of the respective treatment groups *vs*. control; *$p < 0.05$; two-way ANOVA followed by Bonferroni *post-hoc* correction. (**D**) All THC preparations (10 μM) eliminated the mitochondrial membrane potential. Data are after 1h exposure and were expressed as means ± s.d. Colored circles denote independent biological replicates. **$p < 0.05$ *vs*. control; two-way ANOVA for (B,C) and one-way ANOVA for (D), both followed by Bonferroni's *post-hoc* comparison.

Downloadable spreadsheets to figures 1-S9. *Read-out files from GraphPad calling out statistical differences for corresponding figure panels and supplementary figure panels.* Note particular details of ANOVAs and homogeneity testing. Microsoft Excel spreadsheets titled '*Figure 2, P48 proteomics, statistics*.xls' and '*Figure 2, P120 proteomics, statistics*.xls' contained the analytical workflow that led to the results shown in Figures 2C and D.

Supporting Table S1 **-** Immunoreagents used for histochemistry and Western blotting.

Target	Source	Host	Dilution	App.
Hoechst 33,342	Sigma; #23491-52-3	synthetic	1:10,000	IHC
Cleaved Caspase 3 (Asp175)	Cell Signaling; #9661	rabbit	1:500	IHC
c-Fos (Ab-5)	EMD Millipore; #PC38 (2700487)	rabbit	1:1,000	IHC
Parvalbumin	Sigma; #P3088	mouse	1:2,000	IHC
Somatostatin	GeneTex; #GTX71935	mouse	1:1,000	IHC
β-III-Tubulin	Promega; #G7121 (0000161930)	mouse	1:2,000	WB
TOM20 (FL-145)	Santa Cruz; #sc-11415 (J2815)	rabbit	1:500	WB/IHC
Oxphos Cocktail	Abcam; #ab110413 (K2342)	mouse	1:250	WB

Hoechst 33,342 was used as nuclear counterstain. Lot numbers where available are noted behind the catalog number in brackets. *Abbreviations*: IHC, immunohistochemistry; WB, Western blotting.

Supplementary references

1. Tortoriello G, Morris CV, Alpar A, Fuzik J, Shirran SL, Calvigioni D, et al. Miswiring the brain: Delta9-tetrahydrocannabinol disrupts cortical development by inducing an SCG10/stathmin-2 degradation pathway. *EMBO J.* 2014;33(7):668-85.
2. Freeman TP, Groshkova T, Cunningham A, Sedefov R, Griffiths P, and Lynskey MT. Increasing potency and price of cannabis in Europe, 2006-16. *Addiction.* 2019;114(6):1015-23.
3. Hillard CJ, Edgemond WS, and Campbell WB. Characterization of ligand binding to the cannabinoid receptor of rat brain membranes using a novel method: application to anandamide. *J Neurochem.* 1995;64(2):677-83.
4. Bradford MM. A rapid and sensitive method for the quantitation of microgram quantities of protein utilizing the principle of protein-dye binding. *Anal Biochem.* 1976;72:248-54.
5. Mate Z, Poles MZ, Szabo G, Bagyanszki M, Talapka P, Fekete E, et al. Spatiotemporal expression pattern of DsRedT3/CCK gene construct during postnatal development of myenteric plexus in transgenic mice. *Cell Tissue Res.* 2013;352(2):199-206.
6. Calvigioni D, Mate Z, Fuzik J, Girach F, Zhang MD, Varro A, et al. Functional Differentiation of Cholecystokinin-Containing Interneurons Destined for the Cerebral Cortex. *Cereb Cortex.* 2017;27(4):2453-68.
7. Harkany T, Hartig W, Berghuis P, Dobszay MB, Zilberter Y, Edwards RH, et al. Complementary distribution of type 1 cannabinoid receptors and vesicular glutamate transporter 3 in basal forebrain suggests input-specific retrograde signalling by cholinergic neurons. *Eur J Neurosci.* 2003;18(7):1979-92.
8. Susaki EA, Tainaka K, Perrin D, Kishino F, Tawara T, Watanabe TM, et al. Whole-brain imaging with single-cell resolution using chemical cocktails and computational analysis. *Cell.* 2014;157(3):726-39.
9. Walf AA, and Frye CA. The use of the elevated plus maze as an assay of anxiety-related behavior in rodents. *Nat Protoc.* 2007;2(2):322-8.
10. Shirran SL, and Botting CH. A comparison of the accuracy of iTRAQ quantification by nLC-ESI MSMS and nLC-MALDI MSMS methods. *J Proteomics.* 2010;73(7):1391-403.
11. Keimpema E, Barabas K, Morozov YM, Tortoriello G, Torii M, Cameron G, et al. Differential subcellular recruitment of monoacylglycerol lipase generates spatial specificity of 2-arachidonoyl glycerol signaling during axonal pathfinding. *J Neurosci.* 2010;30(42):13992-4007.
12. Miklosi AG, Del Favero G, Bulat T, Hoger H, Shigemoto R, Marko D, et al. Super-resolution Microscopical Localization of Dopamine Receptors 1 and 2 in Rat Hippocampal Synaptosomes. *Mol Neurobiol.* 2018;55(6):4857-69.
13. Antonovaite N, Beekmans SV, Hol EM, Wadman WJ, and Iannuzzi D. Regional variations in stiffness in live mouse brain tissue determined by depth-controlled indentation mapping. *Sci Rep.* 2018;8(1):12517.
14. Chen CY, Caporizzo MA, Bedi K, Vite A, Bogush AI, Robison P, et al. Suppression of detyrosinated microtubules improves cardiomyocyte function in human heart failure. *Nat Med.* 2018;24(8):1225-33.
15. Mason DE, Collins JM, Dawahare JH, Nguyen TD, Lin Y, Voytik-Harbin SL, et al. YAP and TAZ limit cytoskeletal and focal adhesion maturation to enable persistent cell motility. *J Cell Biol.* 2019;218(4):1369-89.
16. Field JS, and Swain MV. Determining the Mechanical-Properties of Small Volumes of Material from Submicrometer Spherical Indentations. *J Mater Res.* 1995;10(1):101-12.
17. Mattei G, Looze N, and Breel EJ. Measuring micro-mechanical properties of (bio)materials by nano-indnetation. *Optics11 Piuma White Paper.* 2017:1-10.
